# Molecular and cellular mechanisms of aging in hematopoietic stem cells and their niches

**DOI:** 10.1186/s13045-020-00994-z

**Published:** 2020-11-23

**Authors:** Lei Zhang, Ryan Mack, Peter Breslin, Jiwang Zhang

**Affiliations:** 1 Department of Cancer Biology, Oncology Institute, Cardinal Bernardin Cancer Center, Loyola University Medical Center, Maywood, IL 60153 USA; 2 Departments of Molecular/Cellular Physiology and Department of Biology, Loyola University Medical Center and Loyola University Chicago, Chicago, IL 60660 USA; 3 Department of Pathology, Loyola University Medical Center, Maywood, IL 60153 USA

**Keywords:** HSCs, Aging, Replication stress

## Abstract

Aging drives the genetic and epigenetic changes that result in a decline in hematopoietic stem cell (HSC) functioning. Such changes lead to aging-related hematopoietic/immune impairments and hematopoietic disorders. Understanding how such changes are initiated and how they progress will help in the development of medications that could improve the quality life for the elderly and to treat and possibly prevent aging-related hematopoietic diseases. Here, we review the most recent advances in research into HSC aging and discuss the role of HSC-intrinsic events, as well as those that relate to the aging bone marrow niche microenvironment in the overall processes of HSC aging. In addition, we discuss the potential mechanisms by which HSC aging is regulated.

## Background

The primary functions of blood cells are transporting oxygen to tissues by red blood cells (RBCs), antagonizing infections caused by pathogenic agents (macrophages and neutrophils), maintaining coagulatory hemostasis (platelets), and confronting and responding adaptively to internal and external antigenic affronts (T and B lymphocyte-mediated immune defense). In the adult human, ~ 4–5 × 10^11 ^blood cells are lost every day due to cellular aging or damage. To replenish the loss of blood cells, approximately the same numbers of blood cells must be produced from bone marrow (BM) hematopoietic progenitors daily [1–3]. Normal homeostatic multi-lineage blood cell regeneration, including immune-related tissue, is primarily maintained through multipotent hematopoietic progenitors (MPPs), which become exhausted over time, whereas lifetime hematopoiesis and immunity are maintained by self-renewable hematopoietic stem cells (HSCs) that are localized within specialized BM microenvironments called HSC niches [4–9].

Like most other tissue-specific stem cells, HSCs are vulnerable to aging-related stress and gradually lose their self-renewal and hematopoietic regenerative capacities (HRC) [10–12]. The process of aging in HSCs is driven by both cell-intrinsic and extrinsic factors, which lead to a reduction in blood cell production and impairment of immune system function [13–16]. Consequently, elderly populations experience higher incidences of anemia, arterial thrombosis, and myeloid and lymphoid malignancies (such as age-related clonal hematopoiesis, myelodysplastic syndromes, acute myeloid leukemia, chronic lymphocytic leukemia, multiple myeloma and non-Hodgkin's lymphoma) [17–19]. In addition, this population may experience declining adaptive immunity, autoimmunity, vaccine failure, and experience increased innate immune-inflammation and susceptibility to infectious diseases [20–23]. Thus, a more in-depth elucidation of the molecular and cellular mechanisms by which HSC aging are inter-regulated will not only help in the development of medications for treating and preventing age-related hematopoietic and immune disorders, but also provide strategies to advance the quality of life of elderly populations by reducing the burden of dysfunctional hematopoiesis and immunity due to aging [24–26].

Changes in elderly human, monkey, and mouse HSCs compared to younger adults include: (1) increased numbers; (2) compromised HRC; (3) skewed myeloid differentiation at the expense of T/B lymphocytes; [27–31] (4) expansion of functionally defective HSCs; and (5) accumulation of clonal hematopoiesis, specifically, genetically mutant HSCs, due to the selective expansion of age-associated somatic mutant HSCs [10, 11, 13, 32–34]. All of these changes explain the aging-related alterations seen in peripheral blood (PB) and tissues, including: (1) attenuated blood cell regeneration in response to radiation or chemical-induced BM damage; (2) increased percentages of granulocytes and monocytes, which is always accompanied by a reduced percentage of T/B lymphocytes and impaired adaptive immunity; (3) reduced anti-infective activity of neutrophils; and (4) slightly reduced RBC and platelet numbers [16, 35, 36]. It needs to be mentioned that all these conclusions regarding the aging of HSCs were obtained from studies of mouse models, primarily owing to the availability of relatively specific cell surface markers that have permitted superior purification and functional assessment of HSC biology. Nevertheless, almost all of these conclusions have been verified in other species, suggesting conserved mechanisms of HSC aging [37]. Thus, mouse models provide reliable tools to study the parameters of aging in human HSCs, as age-related milestones in the murine hematopoietic system reflect those of humans. C57BL6 mice are commonly used to study HSCs. It is commonly accepted that 2–6 month-old mice are young adults, which correspond to 20–39 year-old humans. Mice which are > 20 months-old are considered to be aged mice, corresponding to 65–70 year-old humans. Almost all conclusions of aged-HSCs from previous studies were acquired by comparing HSCs collected from > 20 month-old mice to HSCs collected from 2–5 month-old mice. We will focus on the data obtained from mouse models in this review.

## Can the same markers for analysis of young HSCs be used for aged HSCs?

Significant advances have been made during the past 4 decades in the identification and purification of HSCs in mice, specifically in C57Bl6 mice. HSCs are fundamentally characterized by specialized molecular markers for purification and serial transplantation to determine their capacity to: (1) generate long-term reconstitution of all blood lineages (multipotency) in irradiated recipient animals; and (2) produce multipotent HSCs themselves in a process called self-renewal [38–40]. However, all markers currently in use for HSC analysis were developed using young HSCs, and we should first determine whether these markers can be reliably used when studying old HSCs.

### Development of markers for HSC analysis and purification

Lineage^−^Sca1^+^c-Kit^+^ (LSK) was the first identified constellation of markers for HSC analysis [41], which label cells containing HSCs and all 4 types of MPPs (MPP1, 2, 3 and 4) [42, 43]. These HSCs give rise to MPPs, which in turn produce myeloid or lymphoid committed progenitors that subsequently produce all PB and immune cells. Although MPPs have short-term multi-lineage hematopoietic reconstitutive activity (ST-HRC), each MPP type exhibits lineage bias, suggesting that they possess a lineage-primed feature. Only ~ 2% of LSK cells have LT-HRC, suggesting that approximately 1 in 50 LSK cells are functional HSCs (fHSCs) [44]. Many different combinations of markers have been developed to further separate HSCs from MPPs by the addition of more markers in the staining panel for LSK cells in order to increase the purity of HSCs. For example, the addition of Flk2 and CD34 permitted Weismann’s lab to identify HSCs within the LSKCD34^−^flt3^−^ population [45–47]. By incorporating CD48 and CD150, Morrison’s lab was able to identify HSCs within the LSKCD48^−^CD150^+^ population [48]. By the incorporation of EPCR, Mulligan’s lab identified HSCs within the LSKCD34^−^flt3^−^EPCR^+^ population [49, 50]. The use of Hoechst 33342 staining gave Goodell’s lab the ability to identify HSCs within a side population, (SP)-LSK cells [51, 52]. Such combinations of markers identified particular HSCs called phenotypic HSCs (pHSCs), which are enriched among fHSCs at 20%–40% purity as validated by serial dilution-competitive transplantation assays. HSCs identified by these different combinations of markers show 70–80% overlap, suggesting that in addition to marking fHSCs, each panel of markers also identifies a distinct subset of MPPs. For example, SP-LSK cells are 100% CD34^−/low^Flk-2^−^CD48^−^, and 90% with EPCR^+^ which can be further separated into CD150^hi^, CD150^lo^ and CD150^−^ sub-populations [53]. Thus, further combinations of these markers allowed for increased purity of HSCs [42, 43]. In addition, during the past several years, many HSC-specific reporter mouse lines were generated by inserting a fluorescent protein gene under the control of HSC-specific gene regulators such as Hoxb5-mcherry [54], Fgd5-ZsGr, [55] α-catulin-GFP, [56] Tie2-eGFP [57], and Pdzk1ip1-GFP [9]. In these reporter lines, the fluorescent protein specifically marks all fHSCs in young mice, which comprise 10–35% of pHSCs. Transplantation studies suggested that fluorescent-protein^+^ LSK cells are nearly 100% pure fHSCs that have long-term multi-lineage (LT-ML) HRC. Thus, these HSC reporter mouse strains provide useful, reliable tools for HSC studies.

### Increase in phenotypic HSC number and decrease in regenerative capacity during aging

**Fig. 1:**
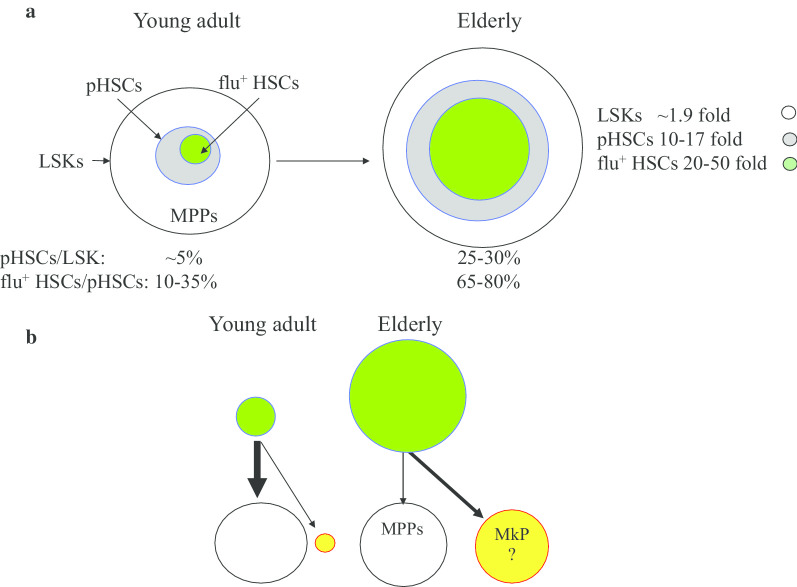
Aging-related changes in pHSCs and flu^+^ HSCs. **a** Compared to young mice, LSK cells are increased by 1.9-fold in BM of old mouse pHSCs. However, HSCs and flu^+^HSCs in BM of old mice are expanded by 10–17-fold and 20–50-fold, respectively, owing to the dramatically increased ratios of pHSCs/LSK and flu^+^HSCs/pHSCs. **b** The reduction in ratios of pHSCs/LSK and flu^+^HSCs/pHSCs in old mice might be due to the reduced production of MPPs and/or increased production of MKPs or other committed progenitors which bypass the MPP stage

Compared to young adult mice, elderly mice display a significant increase in the number of pHSCs (~ 17-fold for CD34^−^ LSK cells, [27] ~ 15-fold for LSK-CD48^−^CD150^+^, ~ 12-fold for CD34^−^LSK-CD48^−^CD150^+^EPCR^+^, [58] ~ 5-fold for SP-LSK [52 ]and ~ 10-fold for CD34^−^LSK-CD48^−^CD150^+^FLK2^−^) [59]. Nevertheless, transplantation studies suggested that the LT-ML HRC of the unpurified BM cells isolated from old mice is increased by only ~ twofold compared to young mice, which is consistent with the ~ 2-fold increase in functional HSCs determined by standard serial dilution and competitive transplantation assays [27, 60]. Interestingly, compared to young adults, the 2-fold increased LT-HRC in old BM cells is better correlated to the ~ 1.9-fold increase in LSK cells [27, 61, 62]. This suggests that all LT-ML HRC cells are enriched within the LSK cell population. However, a 2-fold increase in fHSCs is not consistent with the 10–17-fold increase in pHSCs. Nevertheless, all fHSCs that have LT-ML HRC are still within the pHSC population among old mice, regardless of which panel of markers was used, suggesting that fHSCs are enriched within the pHSC population in old mice. The dramatic increase in pHSCs in old mice was confirmed by studies of HSC reporter mice such as Hoxb5-mCherry, Fgd5-ZsGr, Pdzk1ip1-GFP, and Gprc5c-EGFP [54–56, 63]. The percentage of fluorescence^+^ HSCs is consistently increased in pHSCs of old mice (65–80%) compared to the percentage of fluorescence^+^ HSCs in young animals (10–35%) in these reporter lines. Thus, the number of fluorescence^+^ HSCs is increased 25–50-fold in old mice compared to young mice. (Fig. 1a). The increased pHSCs and fluorescence^+^ HSCs in old mice are not the result of the induction of HSC markers in MPPs by inflammatory cytokines in the BM environment of older animals, because the expression of the fluorescent protein in the reporter mice cannot be induced by any of the inflammatory cytokines. Therefore, increased pHSCs and fluorescence^+^ HSCs in old animals are unlikely to be due to increased contamination of MPPs. The markers that have been used to identify HSCs in young adults can still reliably label HSCs in the elderly.

**Fig. 2:**
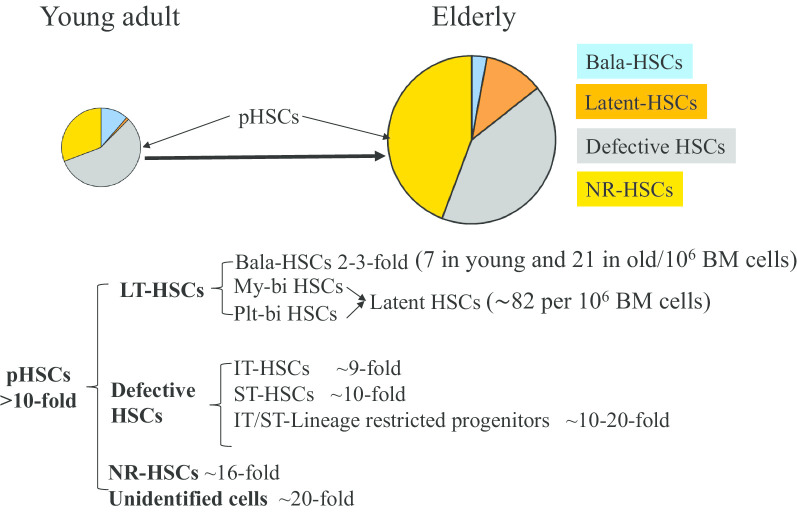
Aging-related changes in fHSCs. Single-cell transplantation studies demonstrate a 2–3-fold expansion of Bala-HSCs, suggesting that functionally normal HSCs exist in aged mice. A significant number of latent HSCs can be detected only in elderly. In addition, more defective HSCs and NR-HSCs can be detected in older mice

Two potential mechanisms explain the inconsistency of pHSCs, fluorescence^+^ HSCs and fHSCs in old animals are the following: (1) if the HSCs are functionally homogeneous, as suggested in the early HSC hierarchical model, then the LT-ML HRC of almost all individual HSCs should be attenuated in older mice; however, (2) if the HSCs are functionally heterogeneous, then significantly more functionally defective HSCs should accumulate in old animals with only a proportion of HSCs still maintaining LT-ML HRC. Almost all recent studies support the second mechanism. The significant increase in the percentage of pHSCs and fluorescence^+^ HSCs within the LSK population in old mice is due to differentiation defects in aged HSCs, which produce fewer MPPs compared to young HSCs. These defects could be either differentiation inhibition or differentiation to committed progenitors, such as megakaryocyte progenitors (MkPs) that bypass the MPP stage (Fig. 2).

## Increase in lineage-biased HSCs and functionally defective HSCs in older animals

Single cell studies demonstrated that HSCs are functionally heterogeneous in terms of both mature cell production and the durability of self-renewal. Compared to young HSCs, such heterogeneity is significantly amplified in aged HSCs as determined by in vitro single cell culturing, in vivo single cell transplantation, and single cell RNA sequencing studies [31, 64–69].

### Significant increase in lineage-biased HSCs and functional defects in HSCs with subtle increase in balanced HSCs in old mice

In in vitro stromal co-cultures, [27, 58] the behaviors of HSCs are not the same in terms of their colony-forming efficiency, cellular components or colony sizes. Most young HSCs generate large, multi-lineage mixed colonies, while only a small proportion of old HSCs are able to generate such large multi-lineage clones. Aged HSCs showed reduced clonogenic efficiency, delayed proliferation and reduced multipotency with myeloid lineage bias as demonstrated by reduced numbers of colonies, longer times to form colonies, and more small-sized uni-lineage or oligo-lineage colonies dominated by granulocytes and monocytes. Such heterogeneous features of HSCs were confirmed by single-cell transplantation studies. Based on myeloid and lymphoid reconstitution ratios, Eaves’ lab classified the LT-HSCs into α- and β- subtypes [66, 70]. Both α- and β- subtypes have robust self-renewal activity and LT-HRC. The α-HSCs are myeloid-biased (My-bi) HSCs that produce blood cells with a lower lymphoid/myeloid ratio due to an inherited deficiency in lymphocyte differentiation [70]. β-HSCs are balanced (Bala)-HSCs, which produce relatively balanced ratios of lymphocytes to myeloid cells. The number of α-HSCs and β-HSCs dynamically changes during development and aging. Compared to young adults, α-HSCs are significantly increased in old animals, whereas β-HSCs are slightly increased [70].

The expression of several megakaryocyte–platelet markers, such as CD150 [31], CD41 [71], CD61 [72] and Vwf, [68] are increased in aged HSCs. In addition, Neogenin-1 (NEO1), a multifunctional transmembrane receptor, is also increased in aged HSCs [59]. By adding such markers to the well-defined pHSC panels, along with transplantation functional studies, several labs were able to largely isolate My-bi HSCs (CD150^hi^, CD41^+^, CD61^+^, c-Kit^hi^, Vwf^int^ or Neo1^+^) from Bala-HSCs (CD150^lo^, CD41^−^, CD61^−^, c-Kit^Int^, Vwf^lo^ or Neo1^−^) [71, 73]. As is the case with α- and β- HSCs, My-bi HSCs are significantly expanded in older animals, whereas Bala-HSCs are slightly increased [70]. However, all these studies used antibodies against the CD45 isoforms CD45.1 and CD45.2 to distinguish blood cells derived from testing HSCs from blood cells derived from competitor cells and host cells. Because CD45 cannot be detected in both RBCs and platelets, all these studies only examined granulocyte, monocyte and lymphocyte contributions to total HSCs but failed to detect RBC and platelet contributions.

By using HSCs isolated from fluorescent protein^+^ mice, Jacobsen’s lab identified platelet-biased (Plt-bi) HSCs. Such HSCs express high levels of Vwf (Vwf^hi^), which generate either platelets alone or platelets with minimal RBCs and granulocytes. The existence of Plt-bi HSCs was verified by both single-cell RNAseq assay and noninvasive in situ fate mapping methods in unperturbed mouse hematopoiesis [4, 5, 7, 74], suggesting that Plt-bi HSCs are a feature of native hematopoiesis [74, 48, 49]. These studies demonstrated that Plt-bi HSCs represent a proportion of My-bi HSCs. As is the case with My-bi HSCs, Plt-bi HSCs are also significantly expanded in old mice. Studies suggested that at a single-cell level, both Plt-Bi HSC and My-bi HSC produce a lower output of mature blood cells than Bala-HSCs in primary transplantation recipients. However, serial transplantation studies suggest that some Plt-bi HSCs and My-bi HSCs have higher self-renewal capacity and are able to generate Bala-HSCs in the recipient BM [75]. This suggests that Plt-bi HSCs and My-bi HSCs are at the apex of the HSC hierarchy, upstream of Bala-HSCs. However, due to the lack of reliable assays to further separate Plt-bi HSCs from My-bi HSCs, the exact relationship between Plt-bi HSCs and My-bi HSCs has not been delineated. Further study will need to determine whether Plt-bi HSCs are at the apex of the HSC-hierarchy, upstream of both My-bi HSCs and Bala-HSCs, or whether Plt-bi HSCs and My-bi HSCs are at the same level in the HSC hierarchy.

**Fig. 3:**
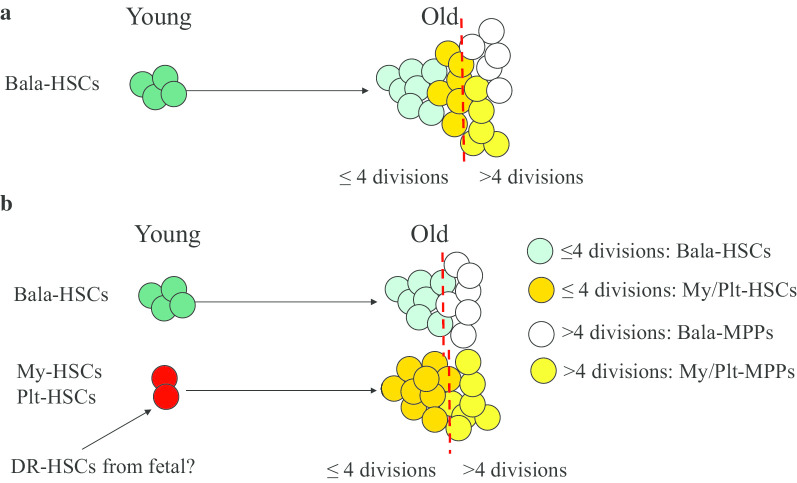
Models for aging-driven expansion of Plt/My-bi HSCs. Two models were proposed to explain the origin of Plt/My-bi HSCs. **a**. The Plt/My-bi HSCs are generated during the Bala-HSC proliferation. **b** Plt/My-bi HSCs are expanded from pre-existing Plt/My-bi HSCs in young adults

By doing a more detailed analysis of a relatively large series of single HSC transplantations, Yamamoto et al. found that Bala-HSCs are expanded 3-fold in old mice, while My-bi/Plt-bi HSCs are expanded > 60 fold. Many of these My-bi/Plt-bi HSCs displayed multipotent output (were able to produce all 5 blood cell lineages) in secondary recipients, suggesting a latent type of HSC (also called MyRP). Such latent HSCs can be identified in BM of old mice but not in young mice [76]. In addition, significantly more functionally defective HSCs, such as intermediate-term and short-term HSCs, were detected in old mice. Furthermore, significantly more non-hematopoietic reconstituting (NR)-HSCs are detected in old mice. Such NR-HSCs failed to reconstitute any of the 5 lineages of blood cells in recipient mice, probably due to a failure of survival, homing or proliferation during transplantation. There is also the possibility that some of the NR-HSCs produce innate immune cells that are localized in tissues and cannot be detected using current strategies. (Fig. 3).

Taken together, HSCs are highly heterogeneous. The increased number of pHSCs in old mice is primarily due to a dramatic increase in My-bi HSCs, Plt-bi HSCs, latent-HSCs and NR-HSCs, with a subtle increase in Bala-HSCs. Importantly, the Plt-bi, My-bi and latent HSCs are less effective in LT-HRC compared to Bala-HSCs, as demonstrated in primary recipients, probably due to their bypassing of the intermediate steps in the generation of mature progeny. Nevertheless, these lineage-biased HSCs are true fHSCs, not short-term HSCs or MPPs, because they are able to produce multi-lineage blood cells in secondary transplant recipients. It also suggests that the ineffective HRC of some of lineage-biased HSCs is induced by BM environmental influences in old mice, which can be reversed when transplanted into a younger BM microenvironment. Functionally defective, aged HSCs are either dormant or lineage-primed, and serial transplantation will be required in order to thoroughly study HSC aging [75].

### What is the origin of lineage-biased HSCs and functionally defective HSCs in old mice?

**Fig. 4:**
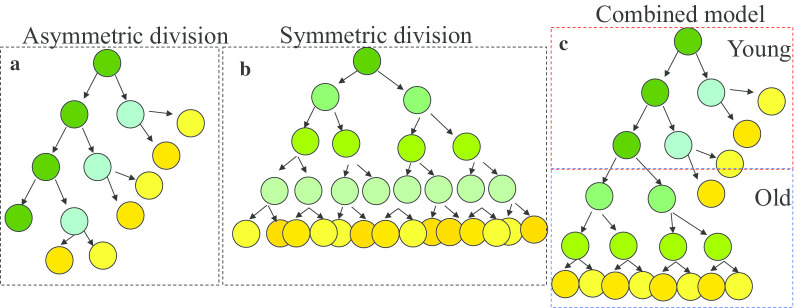
Models for aging-related changes in symmetric and asymmetric division in HSCs. **a** Asymmetric division of HSCs helps to maintain functional HSC numbers by distributing stemness factors to one of the daughter cells and differentiation factors to the other. **b** Symmetric division of HSCs leads to a gradual dilution of their stemness during each division until it is practically absent. **c** More HSCs in young mice undergo asymmetric division; they switch to symmetric division during aging

Increased NR-HSCs in aged mice can be explained by an increase in apoptosis, senescence or homing defects due to the accumulation of DNA damage as well as mitochondria and/or other key cellular organelles becoming defective. However, the precise origin of My-bi and Plt-bi HSCs in old mice is still being debated. Two hypotheses have been proposed to explain the origin of My-HSCs and Plt-Bi HSCs: (1) they might arise from the cell-intrinsic transition of Bala-HSCs to lineage-biased HSCs[29, 31, 77], or (2) they may be generated from the clonal expansion of preexisting fractions of lineage-biased HSCs [72, 78, 79] (Fig. 4).

By tracking the mitotic history of HSCs during physiologic aging using H2B-GFP^+^ long-term-label retaining (LR) assay, Trumpp’s lab found that HSCs might only divide 5 times during the lifespan of a mouse [42]. During each successive division, HSCs progressively acquire myeloid-primed features at the expense of lymphoid differentiation potential and self-renewal capacity [80]. Moore’s lab demonstrated that HSCs undergo symmetric division in the BM niche and gradually acquire My-bi characteristics, which is always companied by an irreversible reduction in self-renewal capacity. The LR-HSCs (≤ 4 divisions) in old mice contain only fHSCs which still preserve multipotency (with a myeloid bias) and self-renewal potential, whereas the H2B-GFP^−^ non-LR-HSCs (> 4 divisions) represent functionally defective HSCs, intermediate HSCs and lineage-restricted progenitors. Thus, non-LR-HSCs have minimal self-renewal and restricted regenerative capacity [81, 82]. Szade’s lab found that Neo1 is a better marker for separating My-bi HSCs from Bala-HSCs. Neo1^−^Hoxb5^+^ Bala-HSCs give rise to Neo1^+^Hoxb5^+^ My-bi HSCs but the reverse transition is rarely observed. Using a paired daughter-cell assay (PDA), Nakauchi’s lab found that Bala-HSCs asymmetrically give rise to Bala-HSCs and Plt-bi HSCs (MkRPs) or My-bi HSCs, during in vitro short-term incubation [67]. These studies suggest that the My-bi HSCs are produced by Bala-HSCs and these undergo irreversible loss of stemness (self-renewal and multipotency) after the fourth division. However, serial transplantation studies suggest that a proportion of Plt-bi HSCs and My-bi HSCs are latent HSCs which have LT-ML HRC in secondary transplantation. Although these latent HSCs are only able to reconstitute very low levels of platelets, RBCs and granulocytes/monocytes in primary recipients, they have significantly strong self-renewal capacity and are able to reconstitute the 5 lineages of hematopoiesis in secondary recipients [76], suggesting that the lineage-biased feature of aged HSCs is reversible. This conclusion was supported by Jacobsen’s lab, which showed that Plt-bi HSCs are located at the apex of the HSC hierarchy and give rise to Bala-HSCs during transplantation [83]. Such a discrepancy among the conclusions of these studies is primarily due to the different research strategies that were employed. The LR-HSCs in Moore’s studies might contain all real fHSCs including Bala-, My-bi and Plt-bi HSCs as defined by Nakauchi’s lab, whereas the non-LR-HSCs only contain ST-HSCs, IT-HSCs and lineage-restricted progenitors. The Neo1^−^Hoxb5^+^ HSCs in Szade’s study are not pure Bala-HSCs, because they express myeloid differentiation-related genes compared to young HSCs. Thus, reliable markers are required to faithfully distinguish Bala-, My-bi- and Plt-bi-HSCs for determining the relationship of these 3 types of HSCs in the hematopoietic hierarchy. Such information is critical not only for a better understanding of the mechanisms of HSC aging and hematopoietic diseases, but also for improving methods for effective ex vivo HSC expansion.

Studies from several other groups suggested that aging drives the change of the clonal composition of the HSC compartment but not individual HSCs. My-bi and Plt-bi- HSCs already exist in young adults and are expanded during aging. The biological and functional similarity of My-bi and Plt-bi HSCs from young and old mice suggest that a clonal expansion of the pre-existing lineage-biased HSCs occurs [67]. These pre-existing lineage-biased HSCs in young adults might be residual fetal HSCs which have been established as developmentally restricted HSCs in neonatal BM. The developmentally restricted HSCs in neonatal BM most likely develop from early erythromyeloid progenitors (EMPs) generated during the secondary wave of embryonic hematopoiesis in the yolk sac, because they continuously produce B1 and γδT innate immune cells [84, 85]. Future studies need to address whether these pre-existing My-bi HSCs and Plt-bi HSCs in young adults develop from yolk sac EMPs or from AGM-definitive HSCs [86, 87].

## Cell-intrinsic mechanism of HSC aging

Increased stress due to cell replication, redox stress, mitochondrial dysfunction, and DNA damage have been defined as hallmarks of HSC aging. Studies have suggested that HSC-intrinsic changes are induced by proliferation. However, whether HSC-intrinsic changes during aging are primarily induced by BM niche changes or are due to niche-independent changes needs to be investigated in the future. In addition, whether aged HSCs regulate the aging of their niches must be evaluated [88].

### Proliferation drives HSC aging

In the adult, almost all HSCs are quiescent with respect to the cell cycle (maintained in G_0_ phase), with only a small proportion of HSCs (< 5%) at any point in time entering into the cell cycle for hematopoietic regeneration. Early studies suggested that HSCs divide on average every 57 days in mice and once every 18 years in humans [89]. By tracking the cell division-related fluorescence dilution of fluorescent protein-labeled HSCs, Trumpp’s and Hock’s groups found that quiescent mouse HSCs take an average of 128 (56–145) days for one division under normal physiological circumstances [42, 90]. Thus, dormant HSCs divide approximately 5 times over the course of mouse’s lifetime. Recent studies suggested that the self-renewal and LT-ML HRC of HSCs are negatively correlated to their divisional history in normal physiological hematopoiesis [82, 91–93]. Therefore, it is speculated that the major driver of HSC aging is proliferation. Sustaining the quiescent state is pivotal for preserving the stemness of adult HSCs by maintaining their low metabolic activity, epigenetic landscape and genomic stability. The increase in HSC number in the elderly is due to the expansion of functionally attenuated HSCs [23]. Several potential mechanisms were proposed to explain how cell divisions attenuate HSC activity, including the induction of replicative stress, DNA damage, epigenetic landscape changes, metabolic stress and shorting telomere length [94–96].

**Fig. 5:**
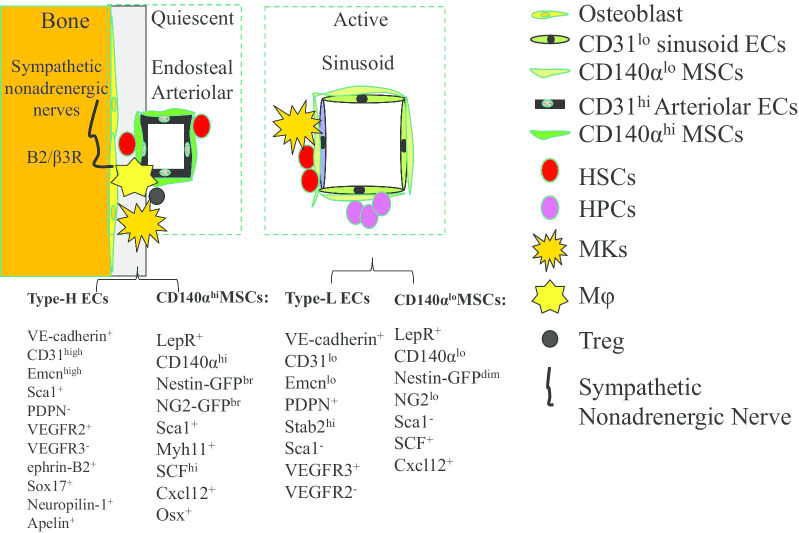
BM HSC niches. Endosteal/arteriolar niches are localized close to the endosteal region of BM, which are populated by CD31^hi^Emcn^hi^ type-H ECs and osteogenic-biased MSC-SCs in the arteriolar capillaries at the distal end of the arterial network (transition zone vessels have substantial branching). Sinusoid niches are localized to the central region of BM and are composed of type-L ECs and adipogenic-biased MSC-SCs. In addition, MKs, Mφ and Treg cells also function as niche cells to maintain HSC quiescence, retain HSCs within their BM niche, and to protect HSCs from immune attack. In addition, the adrenergic sympathetic nerve also functions as niche component for HSCs by regulating HSC relocation between niches

Both symmetric and asymmetric divisions have been proposed for HSC proliferation. In the symmetric division model, HSCs equally distribute their organelles and molecular components into the two daughter cells. In most cases, HSCs undergoing symmetric division will undergo dilution of their stemness and accumulate stress-related damage during each division, eventually becoming exhausted after 4–5 divisions. Only in certain situations, such as fetal HSC development, are both daughter cells able to maintain self-renewal and multipotency leading to an expansion in their number. However, in the asymmetric division model, HSCs unevenly distribute their organelles and molecular components, leading to cellular polarity. The daughter cells which receive healthy organelles, such as mitochondria and lysosomes, [97–100] and self-renewal factors, such as Cdc42, tubulin and H3K36^Ac^, will maintain HSC activity, [11, 30, 101–103] while daughter cells receiving impaired organelles and differentiation factors will lose HSC activity. Thus, HSCs which undergo asymmetric division should be maintained in the HSC pool permanently. The progressive loss of cellular polarity in aged HSCs suggests that more young HSCs undergo asymmetric division and switch to symmetric division in aged HSCs [71, 72]. The increased number of HSCs in old mice might be a compensatory mechanism to overcome their loss of HSC function due to an increase in the frequency of symmetric cell divisions during aging [23] (Fig. 5).

However, some Plt-bi and My-bi HSCs from older animals have more robust self-renewal capacity and can generate Bala-HSCs in transplantation recipients, suggesting that lineage determination can be uncoupled from self-renewal [76, 83, 104]. It was found that HSCs can differentiate into restricted progenitors (including common myeloid, megakaryocyte–erythroid and pre-megakaryocyte progenitors) without undergoing cell division and even before entering S phase of the cell cycle [105]. Therefore, HSC fate decisions can be also uncoupled from physical cell division [105]. The exact mechanism by which proliferation induces HSC aging remains unknown.

### DNA damage and genetic mutations in aging HSCs

Compared to proliferative progenitors which use an error-free homologous recombination (HR) pathway for DNA damage repair, quiescent HSCs are particularly vulnerable to DNA damage due to their preferential use of the error-prone non-homologous end joining (NHEJ) repair pathway [106]. Approximately, a 2- to 3-fold increase in accumulated DNA damage in aged HSCs has been suggested by staining of γH2AX foci, alkaline comet assay, DNA mutation frequency, and LOH assay [77, 94, 107, 108], which explains the acquired mutations, aging-related clonal hematopoiesis and increased risk of myeloid malignancies in the elderly [109–111]. The driver role of DNA damage in HSC aging has been suggested by the premature age-related phenotype of HSCs isolated from mice deficient in DNA repair pathway components [77, 112–114]. DNA damage and mutations in HSCs might be generated by errors in DNA synthesis during DNA replication and/or DNA damage induced by endogenous metabolic factors, such as increased ROS levels or by environmental stresses [115]. DNA damage diminishes HSC function by inducing a DNA damage-repair response through stimulation of cell cycle checkpoint activation, CD53-p21-mediated cell cycle arrest, p16^Ink^ [4]-mediated senescence and p53-PUMA-mediated apoptosis [77, 106, 116]. However, cell-intrinsic factors that cause increased DNA damage in aged HSCs are controversial. Weissman’s lab found that a distinct set of DNA damage repair proteins are reduced in aged HSCs compared to young HSCs [108]. However, Passegué’s lab found an increased number of errors in DNA replication in aged HSCs [94]. Geiger’s lab demonstrated that young and aged HSCs are comparable in DNA damage repair, cell-cycle checkpoint activation and apoptosis, suggesting that increased DNA damage and mutations in aged HSCs are more likely to accumulate gradually [117].

### Replication stress and ribosome biosynthetic stress in HSC aging

Passegué’s lab found that the increased number of γH2AX foci in aged HSCs is not associated with DNA damage due to the lack of co-localization of γH2AX foci with DNA damage proteins (53BP1 and pCHK1) or DNA fragmentation (poly-ADP-ribose (PAR) and TUNEL staining), suggesting that such foci are DNA-damage independent. However, the increase in γH2AX foci is strongly associated with increased staining of the single-stranded DNA binding proteins RPA and ATRIP in proliferative aged HSCs, suggesting a stalled and/or collapsed replication fork-associated γH2AX. The impaired replication of old HSCs is associated with reduced expression of mini-chromosome maintenance (MCM) helicase components. They also observed a long-term persistence of nucleolar γH2AX in quiescent, old HSCs, leading to reduced expression of rRNA owing to ineffective H2AX dephosphorylation due to mislocalization of PP4c. Impaired replication induces the activation of replicative stress in proliferative, aged HSCs and the reduced rRNA expression induces ribosomal biosynthetic stress in quiescent aged HSCs. This study suggests that replicative stress and ribosomal stress are potent drivers of a functional decline in aged HSCs [94]. Consistent with the results of this study, it was found that ribosomal stress is one of the key mechanisms common to diseases related to BM failure [118, 119].

### Mitochondrial and metabolic stress in aging HSCs

Although quiescent HSCs have a relatively high concentration of mitochondria compared to hematopoietic progenitors, mitochondria in HSCs are relatively inactive (morphologically small, round and polarized with low numbers of swollen cristae) [120, 121]. In contrast to hematopoietic progenitors in which mitochondria primarily use the oxidative phosphorylation (OXPHOS) mechanism to generate energy, HSCs use mitochondria-independent glycolysis to satisfy their energy requirements [122]. Although glycolysis generates lower amounts of ATP, such is sufficient for the low bioenergy needs of quiescent HSCs. Studies suggest that maintaining mitochondrial health and low metabolic activity is critical for HSC function, [123–125] and is tightly linked with the cell cycle state of quiescence [126]. Mitochondrial biosynthesis is stimulated in order to meet increased energy demands when transitioning from G_0_ to G_1_. Thus, the proliferation of HSCs is always associated with the glycolysis-to-OXPHOS metabolic switch, epigenetic landscape changes and increased production of ROS [124, 127–131]. ROS activate mitochondrial biosynthesis and protein translation by inducing the Akt-mTor-PGC-1α/β signaling pathway, which leads to HSC differentiation [132–138]. In addition, high levels of ROS cause irreversible damage to mitochondria, lipids, proteins and DNA [139]. Much of the damage to mitochondria in HSCs is irreversible. Thus, HSCs employ several surveillance programs to maintain and/or restore their mitochondria and a low metabolic state in order to preserve their function. For example, the quality of mitochondria in HSCs is regulated by fission/fusion and mitophagy-mediated mitochondrial quality control systems which segregate and remove the damaged mitochondria using lysosomal degradation [57, 140, 141]. However, if the damaged mitochondria cannot be fully removed, then HSCs will distribute the damaged mitochondria to one of the mitotic daughters in order to assure that the healthy mitochondria present in the other daughter cell will preserve the number and function of HSCs [98, 101, 102]. In addition, the signaling pathways which mediate nuclear-mitochondrial communication, including NAD^+^-Sirt1-HIF-1α, Sirt7-NRF1 and Foxo/Sirt3-antioxidants (such as SOD2), also play critical roles in maintaining the low metabolic and inactive states of mitochondria by regulating redox reactions and the mitochondrial unfolding protein response (UPR) [124, 126, 129–131, 142, 143]. Genetic studies suggested that disruption of any of these quality control programs and signaling pathways in mitochondria will induce a premature aged phenotype in HSCs in animals [144]. Compared to young HSCs, down-regulation of Sirt1, Sirt3 and Sirt7 genes, reduced NAD levels, a decline in mitochondrial-encoded genes, and increased Akt-mTor signaling activity are detected in aged HSCs and are associated with increased mitochondrial protein folding stress, ROS levels and increased symmetric division. Finally, overexpression of Sirt3 or Sirt7, raising nuclear NAD^+^ levels or inhibition of either mTor or Cdc42, restore functionality to or even rejuvenate aged HSCs [101, 102, 135, 145, 146].

### Epigenetic deregulation of HSCs during aging

Self-renewal and multipotency are sustained in HSCs by epigenetic machinery that allows for regulation of the epigenetic landscape, including DNA methylation patterns and histone methylation/acetylation profiles, through maintenance of self-renewal gene expression and repression of differentiation and lineage-determining genes [147–151]. The critical role of epigenetic regulators in promoting proper HSC function is well-documented in knockout and transgenic animal models [149, 150, 152–154]. For example, Dnmt1 is a DNA methyltransferase enzyme which re-establishes existing DNA methylation patterns during cellular replication by recognizing hemi-methylated DNA and copying pre-existing DNA methylation marks from the parental template strand to the daughter strand [149]. Dnmt3a/3b acts as a de novo DNA methyltransferase which establishes the new DNA methylation patterns during development and stem cell differentiation [152, 155–157]. HSCs in mice decline in both number and function shortly after deletion of the *Dnmt1* gene, [149, 150, 158] while HSCs are expanded with enhanced self-renewal in *Dnmt3a*-knockout mice and are further enhanced in *Dnmt3a/3b* double-knockouts [152, 156, 157]. Ten-eleven translocation (Tet) methylcytosine dioxygenases catalyze the hydroxylation of DNA 5-methylcytosine (5mC) to 5-hydroxymethylcytosine (5hmC) [159]. *Tet2* knockout promotes self-renewal and expansion of HSCs [160–163]. Polycomb repressor complex 1 (PRC1) and PRC2 repress the expression of target genes by deposition of the repressive marks H2AK119^ub^ [1] and H3K27^Me^ [3] [164, 165]. Mice with deletions of the key components of PRC1 or PRC2, such as Bmi, Ezh1 and Eed, experience HSC exhaustion [166–171]. The H3K4 demethylases Kdm5b (Jarid1b) and Kdm1a (Lsd1), as well as H3K27 demethylases Kdm6a (UTX) and H3K9 methyltransferase SUV39H1, also play essential roles in the regulation of HSC function [172–175]. In addition, histone lysine acetyltransferases Kat6a (Moz), Kat6b (Morf) and Kat8 (Mof) regulate target gene expression by depositing H3K9^ac^, H3K23^ac^/H3K14^ac^ and H4K16^ac^, respectively, on the regulatory regions of target genes. Genetic inactivation of any of these histone acetyl-transferases causes HSC exhaustion in mice [176–179].

Accumulated evidence suggests that HSC aging is regulated by changes in the epigenetic landscape. Comparative studies of epigenetic profiling of young and aged HSCs reveal a number of epigenetic differences (age-related epigenetic drift) that underlie the heterogeneous behavior, lineage-biased feature and clonal expansion of HSCs, as well as an increased risk of leukemic transformation [159, 180, 181, 186, 187, 182]. Compared to young HSCs, there is generally a stable or slight global gain of DNA methylation and a reduction of 5-hmC in old HSCs [159, 183]. However, a substantial proportion of differentially altered DNA methylated regions (DMRs) in aged HSCs is associated with PRC2 target genes (with CpG islands), most of which are positive cell cycle regulators and lineage determining factors. These include increased methylation on the genomic loci associated with lymphoid and erythroid lineages and reduced methylation on the genomic loci associated with the myeloid lineage [159]. Although such epigenetic alterations influence changes in gene expression that are associated with self-renewal and myeloid differentiation of aged HSCs, they contribute to an aging-related functional decline and myeloid differentiation skewing of aged HSCs by regulating gene expression in their differentiated progeny [71, 82, 184–186]. Compared to young HSCs, there is a reduction in H4K16^Ac^ levels and a more widespread distribution of H3K4^me^ [3] and H3K27^me^ [3] in aged HSCs [101]. Most importantly, the aging-related epigenetic changes of HSCs are associated with a proliferative history, suggesting a proliferation-driven epigenetic memory loss [184]. Proliferation drives HSC aging by triggering the switch of HSCs from dormancy and multipotency to cellular activation and lineage priming through inducing an epigenetic switch (for example, a switch from Ezh1-to-Ezh2 PRC2), [82] downregulating DNA methylation regulators such as Dnmt1, Dnmt3b and 3 Tet enzymes, as well as key chromatin modulators such as Bmi, Suz12, Eed, Kat6b, Jarid1b, Suv39H1 and Sirt1 [82, 92, 148, 159, 187]. In addition, mutations in epigenetic modifiers are frequently detected in healthy elderly individuals and these also contribute to epigenetic landscape changes and the physiological process of aging in HSCs [187]. Consistently, obvious changes in epigenetic chromatin modifications were detected in aged HSCs. The expression of the microRNA miR-125b, a regulator of HSCs, is reduced in aged HSCs in both human and mouse. miR-125b represses the expression of histone methyltransferase SUV39H1 leading to a global reduction in H3K9^Me^ [3] and loss of heterochromatin structure. Overexpression of miR-125b and inhibition of SUV39H1 in young HSCs induces loss of B cell potential, [175] while inhibition of miR-125b and upregulation of SUV39H1 in old HSCs promotes B cell potential [175].

By comparing gene expression profiling, the DNA methylation landscape and histone modification patterns in parallel within purified HSCs from old mice and young mice, Goodall’s lab found that there are not only more H3K4^me^ [3] peaks but also broader H3K4^me^ [3] peaks across HSC identity and self-renewal genes. Also observed was an increase in DNA methylation at transcription factor binding sites associated with differentiation-promoting genes in aged HSCs. Gene expression profiling demonstrates reduced TGF-β signaling and increased rDNA expression/ribosome activity in aged HSCs. This study suggests that epigenetic changes in aged HSCs might reinforce self-renewal and antagonize differentiation [159]. The discrepancy between the results of this study and other studies might be due to the more purified state of HSCs that were used in the latter study. The reinforced self-renewal epigenetic landscape changes in aged HSCs suggested by this study might reflect the enhanced self-renewal potential of Plt-bi and My-bi HSCs observed in old animals, while the impaired self-renewal and lineage-biased epigenetic changes in aged HSCs detected by other studies might be due to contamination by functionally defective HSCs and MPPs during analysis.

## Age-related HSC niche changes

Although the Bala-HSCs in old mice express many myeloid and platelet genes, they retain normal lymphoid commitment potential when transplanted into a young BM microenvironment [31, 59, 188, 189]. Even some Plt-/My-bi HSCs regain lymphoid differentiation potential upon transplantation into young mice. These reversibility features of aged HSCs in the BM niches of young recipients suggest that the Plt-/My-biased commitment of aged HSCs is largely stimulated by the niche environment. HSC niches also undergo aging-related structural and functional changes which induce inflammatory challenges to HSCs [72]. Therefore, we might be able to rejuvenate aged HSCs by improving niche cell function and repressing inflammation in BM [12, 190].

### HSC niches in BM

**Fig. 6:**
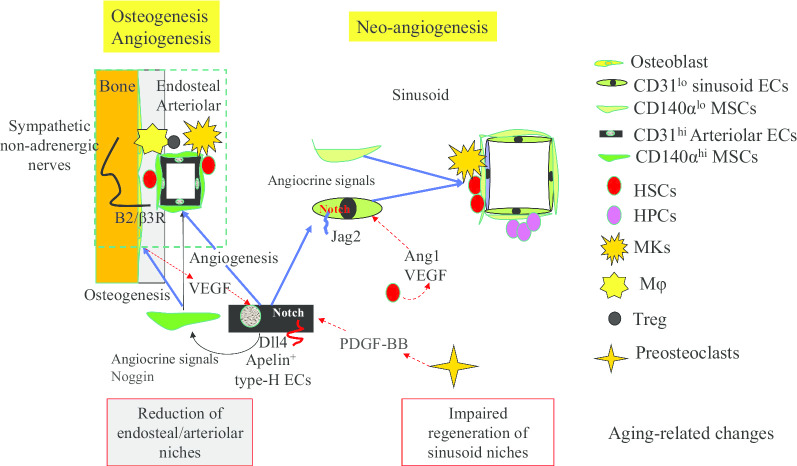
HSC niche regeneration. Type-H ECs in arteriolar niches are stimulated by Dll4-Notch signaling to produce angiocrine factors. Such factors stimulate angiogenesis and osteogenesis to generate endosteal/arteriolar niches during early development and maintain these niches into adulthood. In response to irradiation or chemically induced BM damage, HSCs produce angiopoietin I/VEGF and ECs express Jag2. Such factors collaboratively induce the regeneration of sinusoid niches by stimulating the production of angiocrine factors by type-H ECs. In old mouse BM, the endosteal/arteriolar niches are significantly restricted while the sinusoid niches show minimal changes

HSC niches in BM are composed of multiple hematopoietic and non-hematopoietic cells interacting in a complex 3-dimensional architecture to support HSC function [191]. Three types of BM niches for HSCs have been described, these being designated the endosteal, [192–195] arteriolar, [196] and sinusoid niches (Fig. 6) [54, 56, 197, 198]. However, the specific localization of HSCs and their niches remain difficult to determine and so are still largely unknown [48, 54, 56, 192–203]. Endosteal niches are primarily composed of spider-shaped N-cadherin^+^ osteoblastic (SNO) cells together with an osteopontin-rich extracellular matrix within endosteal tissue. SNO cells are a type of osteogenic mesenchymal stromal cell (MSC) which maintain the quiescent state of hibernating reserve HSCs and protect such HSCs from the lethal effects of cancer chemotherapy [193, 204]. Endosteal niches play such a role probably through Jagged 1-stimulated Notch signaling and osteopontin-mediated proliferative repression in HSCs [192, 205–208]. In addition, lymphoid-bi-HSCs or B-cell progenitors might be primarily localized to osteoblastic niches. Osteoblastic MSCs, together with perivascular BM MSCs, support B-lymphopoiesis by producing lymphocyte-specific cytokines such as Cxcl12 and IL-7 [209–213]. Furthermore, T-reg cells in endosteal niches provide an immune-privileged microenvironment to protect HSCs from immune attack [214–216]. Both arteriolar and sinusoid niches are composed of vascular endothelial cells (ECs) and surrounding MSCs [197, 203, 217]. However, the ECs and MSCs in arteriolar and sinusoid niches are not the same based on their cell surface markers, gene expression patterns and cytokine profiles, suggesting that they are potentially functionally different [218]. For example, the ECs in arteriolar niches are VE-cadherin^+^CD31^high^Emcn^high^Sca1^+^VEGFR2^+^VEGFR3^−^ephrin-B2^+^Sox17^+^Neuropilin-1^+^ type-H ECs, while the ECs in sinusoid niches are VE-cadherin^+^CD31^low^Emcn^low^Stab2^high^Sca1^−^VEGFR3^−^VEGFR2^+^ type-L ECs. The MSCs in arteriolar niches are LepR^+^CD140α^high^Nestin-GFP^bright^NG2-GFP^bright^Sca1^+^Myh11^+^SCF^high^Cxcl12^+^ osteo-biased MSCs, whereas MSCs in sinusoid niches are LepR^+^CD140α^high^Nestin-GFP^dim^NG2^low^Sca1^−^SCF^+^Cxcl12^+^ adipo-biased MSCs which are distributed throughout the BM [56, 196, 198, 219–222]. Most arteriolar niches are primarily localized in epiphyseal/metaphyseal BM and the endosteal region of diaphyseal BM, which might have certain overlaps with endosteal niches. The type-H ECs in arteriolar niches regulate both angiogenesis and osteogenesis during development. Kunisaki et al*.* reported that in young adult BM, most quiescent HSCs are closely associated with arterioles. It was proposed that arteriolar niches are characterized by low oxygen concentration that helps to maintain quiescence in HSCs for hematopoietic preservation, [198, 223] while sinusoid niches maintain their activated HSCs for active hematopoietic regeneration [196]. However, several recent studies suggested that both quiescent and activated HSCs are localized in sinusoid niches and are almost uniformly in contact with vascular VE-cadherin^+^ ECs and LepR^+^ MSCs [54, 56]. Such a discrepancy might be due to the different markers used to detect HSCs, which could have led to the selective investigation of different subsets of HSCs in the different studies.

In addition, macrophages (Mφ), megakaryocytes (MK) and sympathetic neurons have also been described as component cells of HSC niches. CD169^+^, DARC/CD234^+^ and/or αSMA^+^ Mφ are required for retaining quiescent HSCs in Nestin^+^ endosteal/arteriolar niches. Mφ play such a role by stimulating HSC niche chemokine/cytokine production, CD82-mediated signaling, Cox2-PGE-2-dependent HSC survival and clearance of senescent CD62L^low^CXCR4^high^ neutrophils [224–229]. Mφ depletion promotes HSC egress into the bloodstream [224–229]. Sympathetic nerves regulate HSC mobilization and circadian oscillation release by stimulating β_2_ or β_3_ adrenergic receptors (AR) in BM MSCs and osteoblasts in collaboration with Mφs and neutrophils [230–233]. Furthermore, MKs spatially associate with a subset of HSCs and maintain HSC quiescence in BM niches. MKs play such a role by producing TNF-βCXCL4 and thrombopoietin through collaborating with other niche cells such as non-myelinating Schwann cells. [16–18, 37, 38] Further study has suggested that most vWF^+^ Plt-/My-bi HSCs are localized within sinusoid niches associated with MKs. MKs regulate the proliferation and HRC of vWF^+^ Plt-/My-bi HSCs, while NG2^+^ MSCs in arteriolar niches regulate quiescence and localization of vWF^−^ Ly/Bala-HSCs [104]. Depletion of MKs in the BM leads to the proliferation of dormant HSCs and expands Plt-/My-bi HSCs [104].

### Heterogeneity of BM MSCs and ECs

Like HSCs and HPCs, MSCs in BM are also a mixture of cell populations which are enriched for MSC-stem cells (MSC-SCs) and early progenitors for adipocytes, osteoblasts, chondrocytes, pericytes and fibroblasts as demonstrated by cell surface markers, gene expression profiles and cytokine profile studies at single-cell resolution [234–240]. Only a small proportion of specific MSCs function as HSCs niches. Such types of MSCs have been defined as Nestin-GFP^+^, CD45^−^CD31^−^Sca1^+^CD24^+^, < 10% of LepR^+^ or Pdgfr^+^Sca1^+^CD51^+^ which have the ability to generate CFU-F and mesenspheres in in vitro culture and can differentiate into all lineages of MSCs including adipocytes, osteoblasts, chondrocytes, pericytes and fibroblasts both in vitro and in vivo, suggesting they are MSC-SCs [196, 203, 219, 241–249]. In addition, different types of hematopoietic progenitors (HPCs) such as B lymphocyte progenitors and erythroid progenitors also have their unique niches formed by different types of MSCs, which produce lineage-instructive cytokines [211, 250, 251]. These studies suggest that MSC-SCs producing self-renewal factors function as niche cells for HSCs, while distinct types of MSC-progenitors producing lineage-specific factors function as niches for distinct types of HPCs. Most importantly, like HSCs, the MSC-SCs are also heterogeneous and might function as niches for different sub-types of HSCs [234, 252, 253]. In further support of this notion, it was found that Nestin-GFP^bright^ and Pdgfr^+^Sca1^+^CD51^+^ MSC-SCs promote HSC expansion in vitro by producing the HSC niche-specific cytokines SCF (KitL), SDF1 (Cxcl12), IL7, angiopoietin 1 (angpt 1) and adhesive molecular Vcam1 [241, 243, 250]. This ability of MSC-SCs is attenuated upon differentiation and can be restored by overexpression of 5 genes (Klf7, Ostf1, Xbp1, Irf3 and Irf7) which revitalize MSC-SCs [254].

The critical role of BM ECs in HSC regulation has been well documented. During conditions of homeostasis, specific types of BM ECs are required for maintaining HSCs by producing the key HSC-specific cytokines Cxcl12, SCF and Jagged 1/2. During hematopoietic regeneration and in vitro culture, BM ECs promote HSC expansion by producing soluble and membrane-bound angiocrine factors including SCF, BMP2/4, IGFBP2, CXCL12, Notch ligands, Wnt5a, EGF, and pleiotrophin [220, 255–265]. However, the stem cells and progenitors for ECs are not clearly identified. Whether there are EC-stem cells (EC-SCs) that selectively function as niches for HSCs needs to be determined in the future [248, 249, 266]. A recent study found that Apelin^+^ ECs represent 12.4% ± 1.3% of BM ECs [249, 267]. Such ECs are a subset of type-H arteriolar ECs that express niche factors, including connexin gap-junction proteins, Notch ligands, and pleiotrophin (PTN). Apeln^+^ ECs are required for maintaining HSCs during steady-state hematopoiesis and regulate hematopoietic regeneration in response to myeloablative injury [249]. It should be emphasized here that HSCs also produce factors to regulate their own niches. For example, HSCs produce VEGF-A and angiopoietin which induce vessel regeneration by stimulating Dll4-Notch signaling in type-H ECs for sprouting angiogenesis and vascular niche regeneration [218, 249]. In addition, type-H ECs also release angiocrine factors and osteogenic factors which promote proliferation and differentiation of osteo-MSC-SCs in endosteal/arteriolar niches for vascularization and osteogenesis [218, 221, 249, 266, 268–275]. Thus, it is most likely the case that HSCs, MSC-SCs and EC-SCs are associated during differentiation and their progeny work together as niches for each other [276].

### Aging of HSC niches

**Fig. 7:**
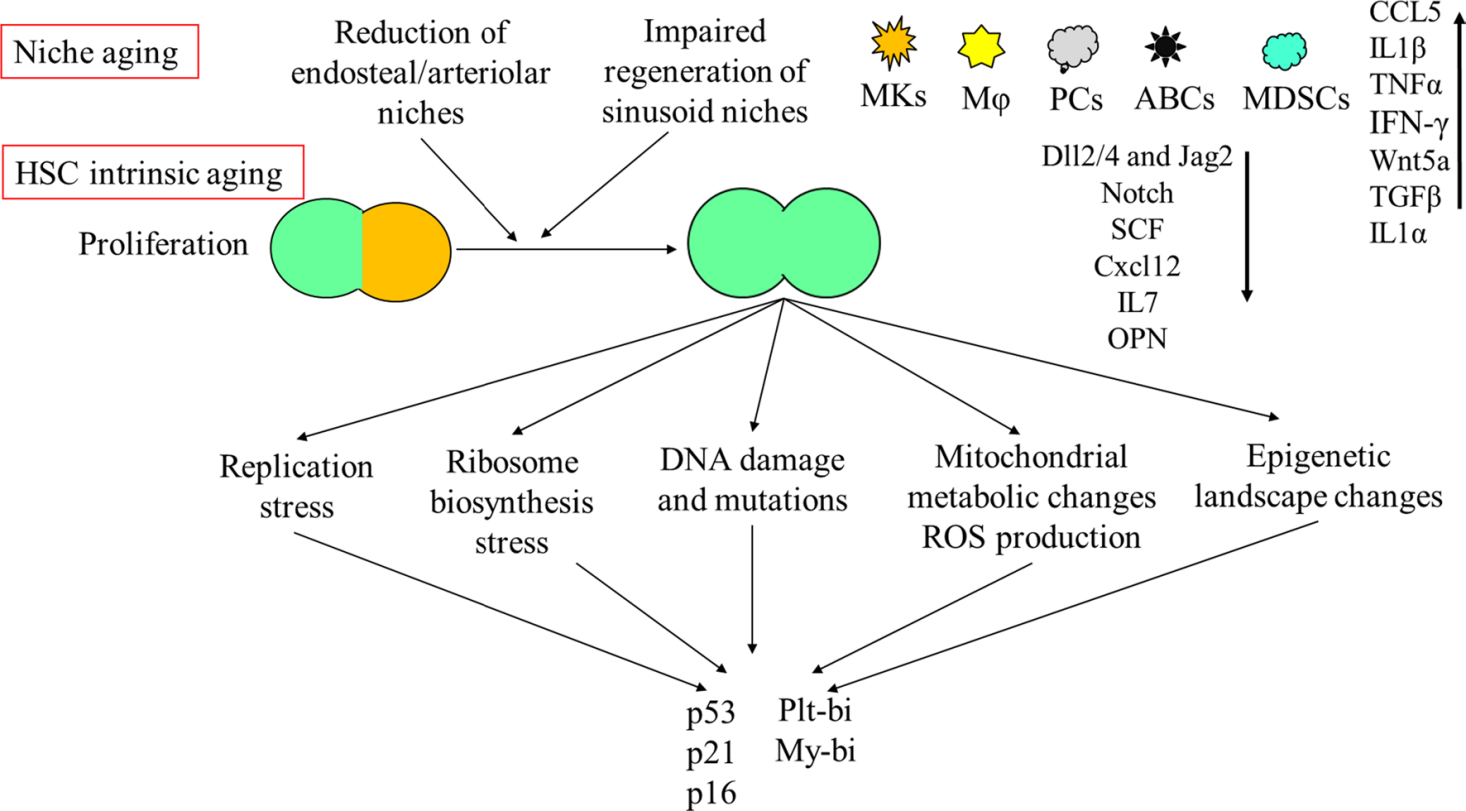
Mechanism of HSC aging. HSCs undergo niche-driven and proliferation-associated changes during aging. Proliferation induces multiple stresses on HSCs including replication, ribosome biosynthesis, DNA damage, as well as metabolic and epigenetic stresses. Such stresses attenuate the self-renewal capacity of HSCs by inducing p53-dependent/independent senescence/apoptosis and promote lineage-biased differentiation by inducing platelet/myeloid genes and repressing lymphoid genes. Aging of HSC niches promotes the switch from asymmetric division to symmetric division in HSCs and impairs the self-renewal of HSCs due to a reduction in key niche factors including SCF, Cxcl12, IL7, and Notch ligands. In addition, the accumulation of MKs, M, plasma cells (PCs), aging-associated B cells, and MDSCs, which is primed by inflammatory mediators, promotes the Plt/My-biased phenotype in HSCs through the production of inflammatory cytokines such as CCL5, IL1β, TNFα, IFN-γ, Wnt5 and TGFβ

HSC niches in BM undergo significant structural and functional alterations during aging [277]. Compared to young mice, although overall vascular density is increased in BM tissue of aged mice, [221, 230] arteries and arterioles are decreased in their length and diameter and the vessels in aged BM display a disorganized orientation. In particular, the vessels containing type-H ECs that bridge the arteriolar and sinusoid capillaries are significantly reduced [278]. However, the sinusoid vasculature is less affected and small capillaries containing type-L ECs (< 6 mm in diameter) in the central BM are expanded [190, 278]. The retraction of endosteal regions and reduced transitional zone vessels in these regions, along with expanded small capillaries in the non-endosteal (central) region, suggest a reduction in endosteal/arteriolar niches with a reduced effect on sinusoid niches [190]. The age-related reduction in endosteal/arteriolar niches is associated with the downregulation of Notch ligands Dll1 and Dll4 in type-H ECs, suggesting aging-related impairment of Notch signaling in the arteriolar niche [236, 278, 279]. In addition, due to the lack of recovery of expression of the endothelial Notch ligand Jag2 after chemotherapy in old sinusoid ECs, such drug treatment causes long-term hematopoietic disruption in the elderly owing to the attenuated recovery of sinusoidal niches [81, 278]. The results of these studies suggest that altered Notch signaling critically contributes to the aging-related defects of both endosteal/arteriolar and sinusoid niches of HSCs [81] (Fig. 7).

Consistent with structural changes in BM niches, the MSCs and ECs in old BM display significant qualitative and quantitative changes. Although the absolute numbers of BM MSCs and ECs are not significantly altered, [280–284] the numbers of Nestin-GFP^bright^PDGFRβ^+^NG2^+^ MSCs and CD31^hi^Emcn^high^ type-H ECs in endosteal regions are reduced in the aged BM, while LepR^+^Nestin-GFP^low^ MSCs and CD31^lo^Emcn^lo^ type-L ECs in the central BM are not reduced [81, 190, 230]. The qualitative changes of MSCs in older BM is demonstrated by: (1) reduced capacity of colony-forming unit fibroblasts (CFU-F) and mesenspheres in vitro; (2) reduced expression of HSC niche factors; [230] and (3) reduced osteogenesis and increased adipogenesis that is associated with lower osteopontin secretion to the extracellular matrix [194, 285]. As a consequence, an increase in adipocytes is a common feature of BM in the elderly and is associated with an increased risk of osteoporosis and bone fractures [247, 286, 287]. Most studies suggested that adipocytes in the BM reduce hematopoietic reconstitution [286–288]. The aging-related contraction of endosteal BM and reduced osteopontin contained in the matrix in the elderly explain the myeloid skewing phenotype owing to the reduction in lymphocyte-specific niches [251, 289–292]. Supporting this idea, it was found that the decline in osteopontin accelerates HSC divisions during aging, and treatment with thrombin-cleaved osteopontin partially reverses the age-associated phenotype of HSCs [194, 254]. The aging-related structural changes of BM niches are also associated with functional declines of vascular ECs as demonstrated by decreased angiogenic potential. Thus, the vascular niche in old mice shows increased leakiness and ROS levels. In addition, ECs and MSCs isolated from older mice show reduced ability to support the expansion of LT-HSCs in co-culture compared to their younger counterparts [293]. It was found that expression of Dll4 in vascular ECs prevents myeloid skewing of HSCs [236].

### Changes in HSC-niche interactions during aging

More HSCs in aged mice are localized at some distance from the endosteal bone surface, arterioles, Nestin-GFP^high^ cells and/or MKs in situ. However, HSC distance from sinusoids and Nestin-GFP^low^ cells appears unchanged [81, 190, 230]. Significantly more HSCs egress into the circulation in old mice [294]. Upon transplantation, aged HSCs display homing defects which correlate with increased HSCs being localized away from the endosteum [101]. Long-term labeling retention assays demonstrate that the most quiescent LR-HSCs with the highest HRC and cellular polarity reside individually and predominantly in Nestin-GFP^low^ perisinusoidal niches in old mice. In contrast, the non-LR-HSCs are largely apolar and were found more frequently in clusters, [278, 279] which are located significantly further away from the vasculature [18, 19]. These studies suggest that due to the contraction of endosteal/arteriolar niches during aging, perisinusoidal niches protect HSCs from aging stresses in the BM of aged mice [81].

In addition to MSCs and ECs, many other types of niche cells, including MKs, Mφ and sympathetic nerves, also undergo significant aging-related changes. For example, the numbers of MKs and Mφ are increased in the BM of old animals; however, the HSC supportive function of these cells has undergone decline in old BM. More HSCs in old animals are localized away from MKs [190, 230] The MKs in aged mice have abundant pseudopodial extensions and fail to maintain HSC quiescence [190]. The aged BM Mφs display impaired ability to clear senescent neutrophils. In addition, aged BM Mφs cause elevated IL-1β and promote the expansion of Plt-/My-bi HSCs at the expense of Bala-HSCs [295]. The response of BM MSCs to adrenergic signaling is altered due to the switch of β_3_-AR to β_2_-AR expression. Such a signaling switch also contributes to HSC aging [296]. Furthermore, age-associated senescent B cells (ABCs), myeloid-derived suppressor cells (MDSCs) and plasma cells are increased in old BM. Both MDSCs and ABCs produce elevated concentrations of pre-inflammatory cytokines like CCL5, IL-1, TNFα and interferon-γ, whereas plasma cells induce the production of inflammatory factors from BM MSCs [297–301]. All of these inflammatory cells also contribute to enhanced myelopoiesis in aging BM [302].

## Conclusions

Significant progress has been made during the last 30 years in the study of aging-related changes in HSCs and their niches. This progress includes: (1) development of reliable panels of antibody markers and genetic animal models for analysis of HSCs in both young and old mice; (2) improved ability for the examination of functional HSCs and determination of the heterogeneity of HSCs by using single-cell transplantation and single-cell sequencing; (3) improved ability to more precisely detect HSC localization in the BM and the structure/components of HSC niches through the use of deep imaging quantification and 3-dimensional microanatomical analysis of the BM niche [303]. Such technical improvements have allowed the examination of the age-dependent structural changes of HSC niches at the resolution of single cells; [221, 278] (4) investigation of the aging-associated accumulation of several types of mature hematopoietic cells and how they impact HSC aging; and (5) identification of potential niche factors and signaling pathways that are associated with HSC aging [268]. All these achievements not only improve our ability to understand the cellular/molecular mechanisms by which physiological aging of the hematopoietic/immunologic system is regulated, but will help us to elucidate the pathogenesis of aging-related hematopoietic disorders.

While all of these foregoing advances are significant, there are still many aspects of this topic that need to be further investigated. First, several conflicting results have been reported with regard to quiescent HSCs, active HSCs and their niches, owing to the different protocols that have been used in the identification of HSCs in situ. For example, some studies used CD11b and CD41 in their lineage cocktails and thus excluded many of the aged HSCs, whereas others did not include these antibodies in their cocktails. Second, the conclusions concerning aging-related changes in HSCs were not always consistent owing to the different standards of purity of HSCs and different pre-conditioning protocols that were used for transplantation functional studies. For example, most research protocols use radiation for pre-conditioning recipient animals in preparation for HSC transplantation. However, it must be kept in mind that this induces significant inflammation and might preferentially stimulate the Plt/My-bi feature in the HSCs being studied. Several recent reported studies used busulfan or anti-Kit antibodies for pre-conditioning of recipient mice, resulting in minimal inflammation [189]. Compared to radiation, HSCs in recipients pre-conditioned by either of the latter two regimens are more balanced in multi-lineage engraftment [189]. Thus, in the future, the Plt-/My-bi feature of aged HSCs needs to be further verified using less inflammatory pre-conditioning assays in order to exclude the possibility that this feature is induced by the inflammatory BM environment in recipients. In addition, different standards were used for evaluating LT-ML HRCs. For example, most previous studies examined only three lineages for engraftment, which failed to detect Plt-bi HSCs. Furthermore, several innate immune cells, such as B1 cells and ILC2P cells, accumulate in old mouse BM as well as in skin/intestinal tissues. Such cells were not considered in previous studies. Whether there are lineage-biased HSCs in BM specific for innate immune cells or not, and whether such HSCs are also increased in aged mice, need to be determined. Lastly, although the heterogeneity of both MSCs and ECs in BM tissue were investigated recently using a single-cell technique, the differentiation hierarchies of MSCs and ECs have not yet been determined. Taken together, the detailed cellular components and mechanisms of HSC niches have not been sufficiently elucidated.

## Data Availability

Not applicable.

## Abbreviations

ABC: Age-associated senescent B cell
AR: Adrenergic receptor
Bala-HSC: Balanced-HSC
BM: Bone marrow
DMR: DNA methylated region
EC: Endothelial cell
EC-SC: EC-stem cell
EMP: Erythromyeloid progenitor
fHSC: Functional HSC
HR: Homologous recombination
HRC: Hematopoietic regenerative capacity
HSC: Hematopoietic stem cell
LSK: Lineage^−^Sca1^+^c-Kit^+^
LT-ML: Long-term multi-lineage
Mφ: Macrophages
MDSC: Myeloid-derived suppressor cell
MK: Megakaryocytes
MkP: Megakaryocyte progenitor
MPP: Multipotent hematopoietic progenitor
MSC: Mesenchymal stromal cell
My-bi: Myeloid-biased
MSC-SC: MSC-stem cell
NR-HSC: Non-hematopoietic reconstituting HSC
NHEJ: Non-homologous end joining
OXPHOS: Oxidative phosphorylation
PAR: Poly-ADP-ribose
PB: Peripheral blood
pHSC: Phenotypic HSC
Plt-bi: Platelet-biased
PRC: Polycomb repressor complex: RBC: red blood cells
PTN: Pleiotrophin
SNO: Spider-shaped N-cadherin+ osteoblastic cell
ST-HRC: Short-term multi-lineage hematopoietic reconstitutive activity
Tet: Ten-eleven translocation

## References

[1] Kaushansky K (2006). Lineage-specific hematopoietic growth factors. N Engl J Med.

[2] Ferretti G, Papaldo P, Cognetti F (2006). Lineage-specific hematopoietic growth factors. N Engl J Med.

[3] Dale DC, Rosenberg PS, Alter BP (2006). Lineage-specific hematopoietic growth factors. N Engl J Med.

[4] Sun J (2014). Clonal dynamics of native haematopoiesis. Nature.

[5] Busch K (2015). Fundamental properties of unperturbed haematopoiesis from stem cells in vivo. Nature.

[6] Chapple RH (2018). Lineage tracing of murine adult hematopoietic stem cells reveals active contribution to steady-state hematopoiesis. Blood Adv.

[7] Pei W (2017). Polylox barcoding reveals haematopoietic stem cell fates realized in vivo. Nature.

[8] Schoedel KB (2016). The bulk of the hematopoietic stem cell population is dispensable for murine steady-state and stress hematopoiesis. Blood.

[9] Sawai CM (2016). Hematopoietic stem cells are the major source of multilineage hematopoiesis in adult animals. Immunity.

[10] Leins H (2018). Aged murine hematopoietic stem cells drive aging-associated immune remodeling. Blood.

[11] deHaan G, Lazare SS (2018). Aging of hematopoietic stem cells. Blood.

[12] Li X (2020). Mechanisms and rejuvenation strategies for aged hematopoietic stem cells. J Hematol Oncol.

[13] Latchney SE, Calvi LM (2017). The aging hematopoietic stem cell niche: Phenotypic and functional changes and mechanisms that contribute to hematopoietic aging. Semin Hematol.

[14] Pang WW, Schrier SL, Weissman IL (2017). Age-associated changes in human hematopoietic stem cells. Semin Hematol.

[15] Chinn IK, Blackburn CC, Manley NR, Sempowski GD (2012). Changes in primary lymphoid organs with aging. Semin Immunol.

[16] Nikolich-Zugich J (2018). The twilight of immunity: emerging concepts in aging of the immune system. Nat Immunol.

[17] Eisenstaedt R, Penninx BW, Woodman RC (2006). Anemia in the elderly: current understanding and emerging concepts. Blood Rev.

[18] Steensma DP (2015). Clonal hematopoiesis of indeterminate potential and its distinction from myelodysplastic syndromes. Blood.

[19] Zink F (2017). Clonal hematopoiesis, with and without candidate driver mutations, is common in the elderly. Blood.

[20] Beerman I, Maloney WJ, Weissmann IL, Rossi DJ (2010). Stem cells and the aging hematopoietic system. Curr Opin Immunol.

[21] Warren LA, Rossi DJ (2009). Stem cells and aging in the hematopoietic system. Mech Ageing Dev.

[22] Gazit R, Weissman IL, Rossi DJ (2008). Hematopoietic stem cells and the aging hematopoietic system. Semin Hematol.

[23] Geiger H, de Haan G, Florian MC (2013). The ageing haematopoietic stem cell compartment. Nat Rev Immunol.

[24] Udroiu I, Sgura A (2019). Rates of erythropoiesis in mammals and their relationship with lifespan and hematopoietic stem cells aging. Biogerontology.

[25] Lian X (2019). RNA-Seq analysis of differentially expressed genes relevant to mismatch repair in aging hematopoietic stem-progenitor cells. J Cell Biochem.

[26] Adelman ER (2019). Aging human hematopoietic stem cells manifest profound epigenetic reprogramming of enhancers that may predispose to leukemia. Cancer Discov.

[27] Sudo K, Ema H, Morita Y, Nakauchi H (2000). Age-associated characteristics of murine hematopoietic stem cells. J Exp Med.

[28] Liang Y, Van Zant G, Szilvassy SJ (2005). Effects of aging on the homing and engraftment of murine hematopoietic stem and progenitor cells. Blood.

[29] Rossi DJ (2005). Cell intrinsic alterations underlie hematopoietic stem cell aging. Proc Natl Acad Sci U S A.

[30] Cho RH, Sieburg HB, Muller-Sieburg CE (2008). A new mechanism for the aging of hematopoietic stem cells: aging changes the clonal composition of the stem cell compartment but not individual stem cells. Blood.

[31] Beerman I (2011). Functionally distinct hematopoietic stem cells modulate hematopoietic lineage potential during aging by a mechanism of clonal expansion. Proc Natl Acad Sci U S A.

[32] Nakamura-Ishizu A, Suda T (2014). Aging of the hematopoietic stem cells niche. Int J Hematol.

[33] Ergen AV, Goodell MA (2010). Mechanisms of hematopoietic stem cell aging. Exp Gerontol.

[34] Konieczny J, Arranz L (2018). Updates on Old and Weary Haematopoiesis. Int J Mol Sci.

[35] Snoeck HW (2013). Aging of the hematopoietic system. Curr Opin Hematol.

[36] Linton PJ, Dorshkind K (2004). Age-related changes in lymphocyte development and function. Nat Immunol.

[37] Yu KR (2018). The impact of aging on primate hematopoiesis as interrogated by clonal tracking. Blood.

[38] Suda T, Arai F, Hirao A (2005). Hematopoietic stem cells and their niche. Trends Immunol.

[39] Weissman IL (2002). The road ended up at stem cells. Immunol Rev.

[40] Eaves CJ (2015). Hematopoietic stem cells: concepts, definitions, and the new reality. Blood.

[41] van de Rijn, M., Heimfeld, S., Spangrude, G.J. & Weissman, I. L. Mouse hematopoietic stem-cell antigen Sca-1 is a member of the Ly-6 antigen family. Proc Natl Acad Sci U S A 86, 4634–4638, doi:10.1073/pnas.86.12.4634 (1989). 10.1073/pnas.86.12.4634 PMC287325 2660142

[42] Wilson A (2008). Hematopoietic stem cells reversibly switch from dormancy to self-renewal during homeostasis and repair. Cell.

[43] Pietras EM (2015). Functionally distinct subsets of lineage-biased multipotent progenitors control blood production in normal and regenerative conditions. Cell Stem Cell.

[44] Okada S (1992). In vivo and in vitro stem cell function of c-kit- and Sca-1-positive murine hematopoietic cells. Blood.

[45] Christensen JL, Weissman IL (2001). Flk-2 is a marker in hematopoietic stem cell differentiation: a simple method to isolate long-term stem cells. Proc Natl Acad Sci U S A.

[46] Yang L (2005). Identification of Lin(-)Sca1(+)kit(+)CD34(+)Flt3- short-term hematopoietic stem cells capable of rapidly reconstituting and rescuing myeloablated transplant recipients. Blood.

[47] Adolfsson J (2001). Upregulation of Flt3 expression within the bone marrow Lin(-)Sca1(+)c-kit(+) stem cell compartment is accompanied by loss of self-renewal capacity. Immunity.

[48] Kiel MJ (2005). SLAM family receptors distinguish hematopoietic stem and progenitor cells and reveal endothelial niches for stem cells. Cell.

[49] Kent DG (2009). Prospective isolation and molecular characterization of hematopoietic stem cells with durable self-renewal potential. Blood.

[50] Balazs AB, Fabian AJ, Esmon CT, Mulligan RC (2006). Endothelial protein C receptor (CD201) explicitly identifies hematopoietic stem cells in murine bone marrow. Blood.

[51] Goodell MA, Brose K, Paradis G, Conner AS, Mulligan RC (1996). Isolation and functional properties of murine hematopoietic stem cells that are replicating in vivo. J Exp Med.

[52] Challen GA, Boles NC, Chambers SM, Goodell MA (2010). Distinct hematopoietic stem cell subtypes are differentially regulated by TGF-beta1. Cell Stem Cell.

[53] Weksberg DC, Chambers SM, Boles NC, Goodell MA (2008). CD150- side population cells represent a functionally distinct population of long-term hematopoietic stem cells. Blood.

[54] Chen JY (2016). Hoxb5 marks long-term haematopoietic stem cells and reveals a homogenous perivascular niche. Nature.

[55] Gazit R (2014). Fgd5 identifies hematopoietic stem cells in the murine bone marrow. J Exp Med.

[56] Acar M (2015). Deep imaging of bone marrow shows non-dividing stem cells are mainly perisinusoidal. Nature.

[57] Ito K (2016). Self-renewal of a purified Tie2+ hematopoietic stem cell population relies on mitochondrial clearance. Science.

[58] Dykstra B, Olthof S, Schreuder J, Ritsema M, de Haan G (2011). Clonal analysis reveals multiple functional defects of aged murine hematopoietic stem cells. J Exp Med.

[59] Gulati GS (2019). Neogenin-1 distinguishes between myeloid-biased and balanced Hoxb5 (+) mouse long-term hematopoietic stem cells. Proc Natl Acad Sci U S A.

[60] Harrison DE (1983). Long-term erythropoietic repopulating ability of old, young, and fetal stem cells. J Exp Med.

[61] Morrison SJ, Wandycz AM, Akashi K, Globerson A, Weissman IL (1996). The aging of hematopoietic stem cells. Nat Med.

[62] Rossi DJ, Bryder D, Weissman IL (2007). Hematopoietic stem cell aging: mechanism and consequence. Exp Gerontol.

[63] Cabezas-Wallscheid N (2017). Vitamin A-retinoic acid signaling regulates hematopoietic stem cell dormancy. Cell.

[64] Wilson NK (2015). Combined single-cell functional and gene expression analysis resolves heterogeneity within stem cell populations. Cell Stem Cell.

[65] Morita Y, Ema H, Nakauchi H (2010). Heterogeneity and hierarchy within the most primitive hematopoietic stem cell compartment. J Exp Med.

[66] Dykstra B (2007). Long-term propagation of distinct hematopoietic differentiation programs in vivo. Cell Stem Cell.

[67] Yamamoto R (2013). Clonal analysis unveils self-renewing lineage-restricted progenitors generated directly from hematopoietic stem cells. Cell.

[68] Sanjuan-Pla A (2013). Platelet-biased stem cells reside at the apex of the haematopoietic stem-cell hierarchy. Nature.

[69] Grinenko T (2014). Clonal expansion capacity defines two consecutive developmental stages of long-term hematopoietic stem cells. J Exp Med.

[70] Benz C (2012). Hematopoietic stem cell subtypes expand differentially during development and display distinct lymphopoietic programs. Cell Stem Cell.

[71] Gekas C, Graf T (2013). CD41 expression marks myeloid-biased adult hematopoietic stem cells and increases with age. Blood.

[72] Mann M (2018). Heterogeneous responses of hematopoietic stem cells to inflammatory stimuli are altered with age. Cell reports.

[73] Shin JY, Hu W, Naramura M, Park CY (2014). High c-Kit expression identifies hematopoietic stem cells with impaired self-renewal and megakaryocytic bias. J Exp Med.

[74] Rodriguez-Fraticelli AE (2018). Clonal analysis of lineage fate in native haematopoiesis. Nature.

[75] Muller-Sieburg CE, Cho RH, Karlsson L, Huang JF, Sieburg HB (2004). Myeloid-biased hematopoietic stem cells have extensive self-renewal capacity but generate diminished lymphoid progeny with impaired IL-7 responsiveness. Blood.

[76] Yamamoto R (2018). Large-scale clonal analysis resolves aging of the mouse hematopoietic stem cell compartment. Cell Stem Cell.

[77] Rossi DJ (2007). Deficiencies in DNA damage repair limit the function of haematopoietic stem cells with age. Nature.

[78] Haas S (2015). Inflammation-induced emergency megakaryopoiesis driven by hematopoietic stem cell-like megakaryocyte progenitors. Cell Stem Cell.

[79] MacLean AL (2017). Single cell phenotyping reveals heterogeneity among hematopoietic stem cells following infection. Stem Cells.

[80] Takizawa H, Regoes RR, Boddupalli CS, Bonhoeffer S, Manz MG (2011). Dynamic variation in cycling of hematopoietic stem cells in steady state and inflammation. J Exp Med.

[81] Sacma M (2019). Haematopoietic stem cells in perisinusoidal niches are protected from ageing. Nat Cell Biol.

[82] Bernitz JM (2020). Memory of divisional history directs the continuous process of primitive hematopoietic lineage commitment. Stem Cell Rep.

[83] Carrelha J (2018). Hierarchically related lineage-restricted fates of multipotent haematopoietic stem cells. Nature.

[84] Waas B, Maillard I (2017). Fetal hematopoietic stem cells are making waves. Stem Cell Investig.

[85] Beaudin AE (2016). A transient developmental hematopoietic stem cell gives rise to innate-like B and T cells. Cell Stem Cell.

[86] Hoeffel G, Ginhoux F (2015). Ontogeny of tissue-resident macrophages. Front Immunol.

[87] Kristiansen TA, Vanhee S, Yuan J (2018). The influence of developmental timing on B cell diversity. Curr Opin Immunol.

[88] Mejia-Ramirez E, Florian MC (2020). Understanding intrinsic hematopoietic stem cell aging. Haematologica.

[89] Cheshier SH, Morrison SJ, Liao X, Weissman IL (1999). In vivo proliferation and cell cycle kinetics of long-term self-renewing hematopoietic stem cells. Proc Natl Acad Sci U S A.

[90] Foudi A (2009). Analysis of histone 2B-GFP retention reveals slowly cycling hematopoietic stem cells. Nat Biotechnol.

[91] Qiu J, Papatsenko D, Niu X, Schaniel C, Moore K (2014). Divisional history and hematopoietic stem cell function during homeostasis. Stem Cell Rep.

[92] Bernitz JM, Kim HS, MacArthur B, Sieburg H, Moore K (2016). Hematopoietic stem cells count and remember self-renewal divisions. Cell.

[93] Sawen, P. et al. Murine HSCs contribute actively to native hematopoiesis but with reduced differentiation capacity upon aging. eLife doi:10.7554/eLife.41258 (2018). 10.7554/eLife.41258 PMC6298771 30561324

[94] Flach J (2014). Replication stress is a potent driver of functional decline in ageing haematopoietic stem cells. Nature.

[95] Kirschner K (2017). Proliferation drives aging-related functional decline in a subpopulation of the hematopoietic stem cell compartment. Cell reports.

[96] Soraas A (2019). Epigenetic age is a cell-intrinsic property in transplanted human hematopoietic cells. Aging Cell.

[97] Loeffler D (2019). Asymmetric lysosome inheritance predicts activation of haematopoietic stem cells. Nature.

[98] Hinge A (2020). Asymmetrically segregated mitochondria provide cellular memory of hematopoietic stem cell replicative history and drive HSC attrition. Cell Stem Cell.

[99] Vannini N (2019). The NAD-booster nicotinamide riboside potently stimulates hematopoiesis through increased mitochondrial clearance. Cell Stem Cell.

[100] Liang R (2020). Restraining lysosomal activity preserves hematopoietic stem cell quiescence and potency. Cell Stem Cell.

[101] Florian MC (2012). Cdc42 activity regulates hematopoietic stem cell aging and rejuvenation. Cell Stem Cell.

[102] Florian MC (2018). Aging alters the epigenetic asymmetry of HSC division. PLoS Biol.

[103] Florian MC (2013). A canonical to non-canonical Wnt signalling switch in haematopoietic stem-cell ageing. Nature.

[104] Pinho S (2018). Lineage-biased hematopoietic stem cells are regulated by distinct niches. Dev Cell.

[105] Grinenko T (2018). Hematopoietic stem cells can differentiate into restricted myeloid progenitors before cell division in mice. Nat Commun.

[106] Mohrin M (2010). Hematopoietic stem cell quiescence promotes error-prone DNA repair and mutagenesis. Cell Stem Cell.

[107] Rube CE (2011). Accumulation of DNA damage in hematopoietic stem and progenitor cells during human aging. PLoS ONE.

[108] Beerman I, Seita J, Inlay MA, Weissman IL, Rossi DJ (2014). Quiescent hematopoietic stem cells accumulate DNA damage during aging that is repaired upon entry into cell cycle. Cell Stem Cell.

[109] Cancer Genome Atlas Research, N. Genomic and epigenomic landscapes of adult de novo acute myeloid leukemia. N Engl J Med 368, 2059–2074, doi:10.1056/NEJMoa1301689 (2013). 10.1056/NEJMoa1301689 PMC3767041 23634996

[110] Genovese G (2014). Clonal hematopoiesis and blood-cancer risk inferred from blood DNA sequence. N Engl J Med.

[111] Welch JS (2012). The origin and evolution of mutations in acute myeloid leukemia. Cell.

[112] Nijnik A (2007). DNA repair is limiting for haematopoietic stem cells during ageing. Nature.

[113] de Boer, J. et al. Premature aging in mice deficient in DNA repair and transcription. *Science* 296, 1276–1279, doi:10.1126/science.1070174 (2002). 10.1126/science.1070174 11950998

[114] Behrens A, van Deursen JM, Rudolph KL, Schumacher B (2014). Impact of genomic damage and ageing on stem cell function. Nat Cell Biol.

[115] Walter D (2015). Exit from dormancy provokes DNA-damage-induced attrition in haematopoietic stem cells. Nature.

[116] Yahata T (2011). Accumulation of oxidative DNA damage restricts the self-renewal capacity of human hematopoietic stem cells. Blood.

[117] Moehrle BM (2015). Stem cell-specific mechanisms ensure genomic fidelity within hscs and upon aging of HSCs. Cell reports.

[118] Ebert BL (2008). Identification of RPS14 as a 5q- syndrome gene by RNA interference screen. Nature.

[119] Dokal I, Vulliamy T (2008). Inherited aplastic anaemias/bone marrow failure syndromes. Blood Rev.

[120] Ito K, Suda T (2014). Metabolic requirements for the maintenance of self-renewing stem cells. Nat Rev Mol Cell Biol.

[121] de Almeida, M. J., Luchsinger, L. L., Corrigan, D. J., Williams, L. J. & Snoeck, H. W. Dye-independent methods reveal elevated mitochondrial mass in hematopoietic stem cells. Cell Stem Cell 21, 725–729 e724, doi:10.1016/j.stem.2017.11.002 (2017). 10.1016/j.stem.2017.11.002 PMC5728653 29198942

[122] Takubo K (2013). Regulation of glycolysis by pdk functions as a metabolic checkpoint for cell cycle quiescence in hematopoietic stem cells. Cell Stem Cell.

[123] Vannini N (2016). Specification of haematopoietic stem cell fate via modulation of mitochondrial activity. Nat Commun.

[124] Rimmele P (2015). Mitochondrial metabolism in hematopoietic stem cells requires functional FOXO3. EMBO Rep.

[125] Sukumar M (2016). Mitochondrial membrane potential identifies cells with enhanced stemness for cellular therapy. Cell Metab.

[126] Mohrin, M. et al. Stem cell aging. A mitochondrial UPR-mediated metabolic checkpoint regulates hematopoietic stem cell aging. Science 347, 1374–1377, doi:10.1126/science.aaa2361 (2015). 10.1126/science.aaa2361 PMC4447312 25792330

[127] Bigarella CL, Liang R, Ghaffari S (2014). Stem cells and the impact of ROS signaling. Development.

[128] Mantel CR (2015). Enhancing hematopoietic stem cell transplantation efficacy by mitigating oxygen shock. Cell.

[129] Yalcin S (2008). Foxo3 is essential for the regulation of ataxia telangiectasia mutated and oxidative stress-mediated homeostasis of hematopoietic stem cells. J Biol Chem.

[130] Miyamoto K (2007). Foxo3a is essential for maintenance of the hematopoietic stem cell pool. Cell Stem Cell.

[131] Miyamoto K (2008). FoxO3a regulates hematopoietic homeostasis through a negative feedback pathway in conditions of stress or aging. Blood.

[132] Liu X (2017). Regulation of mitochondrial biogenesis in erythropoiesis by mTORC1-mediated protein translation. Nat Cell Biol.

[133] Chen C (2008). TSC-mTOR maintains quiescence and function of hematopoietic stem cells by repressing mitochondrial biogenesis and reactive oxygen species. J Exp Med.

[134] Liang R, Ghaffari S (2014). Stem cells, redox signaling, and stem cell aging. Antioxid Redox Signal.

[135] Qian P (2016). The Dlk1-Gtl2 locus preserves LT-HSC function by inhibiting the PI3K-mTOR pathway to restrict mitochondrial metabolism. Cell Stem Cell.

[136] Bigarella CL (2017). FOXO3 transcription factor is essential for protecting hematopoietic stem and progenitor cells from oxidative DNA damage. J Biol Chem.

[137] Ferber EC (2012). FOXO3a regulates reactive oxygen metabolism by inhibiting mitochondrial gene expression. Cell Death Differ.

[138] Yeo H (2013). FoxO3 coordinates metabolic pathways to maintain redox balance in neural stem cells. EMBO J.

[139] Norddahl GL (2011). Accumulating mitochondrial DNA mutations drive premature hematopoietic aging phenotypes distinct from physiological stem cell aging. Cell Stem Cell.

[140] Palikaras K, Lionaki E, Tavernarakis N (2018). Mechanisms of mitophagy in cellular homeostasis, physiology and pathology. Nat Cell Biol.

[141] Ho TT (2017). Autophagy maintains the metabolism and function of young and old stem cells. Nature.

[142] Rimmele P (2014). Aging-like phenotype and defective lineage specification in SIRT1-deleted hematopoietic stem and progenitor cells. Stem Cell Rep.

[143] Qiu X, Brown K, Hirschey MD, Verdin E, Chen D (2010). Calorie restriction reduces oxidative stress by SIRT3-mediated SOD2 activation. Cell Metab.

[144] Luchsinger LL, de Almeida MJ, Corrigan DJ, Mumau M, Snoeck HW (2016). Mitofusin 2 maintains haematopoietic stem cells with extensive lymphoid potential. Nature.

[145] Brown K (2013). SIRT3 reverses aging-associated degeneration. Cell Rep.

[146] Gomes AP (2013). Declining NAD(+) induces a pseudohypoxic state disrupting nuclear-mitochondrial communication during aging. Cell.

[147] Bennett-Baker PE, Wilkowski J, Burke DT (2003). Age-associated activation of epigenetically repressed genes in the mouse. Genetics.

[148] Chambers SM (2007). Aging hematopoietic stem cells decline in function and exhibit epigenetic dysregulation. PLoS Biol.

[149] Trowbridge JJ, Snow JW, Kim J, Orkin SH (2009). DNA methyltransferase 1 is essential for and uniquely regulates hematopoietic stem and progenitor cells. Cell Stem Cell.

[150] Broske AM (2009). DNA methylation protects hematopoietic stem cell multipotency from myeloerythroid restriction. Nat Genet.

[151] Park IK (2003). Bmi-1 is required for maintenance of adult self-renewing haematopoietic stem cells. Nature.

[152] Challen GA (2014). Dnmt3a and Dnmt3b have overlapping and distinct functions in hematopoietic stem cells. Cell Stem Cell.

[153] Challen GA (2012). Dnmt3a is essential for hematopoietic stem cell differentiation. Nat Genet.

[154] Trowbridge JJ (2012). Haploinsufficiency of Dnmt1 impairs leukemia stem cell function through derepression of bivalent chromatin domains. Genes Dev.

[155] Okano M, Bell DW, Haber DA, Li E (1999). DNA methyltransferases Dnmt3a and Dnmt3b are essential for de novo methylation and mammalian development. Cell.

[156] Mayle A (2015). Dnmt3a loss predisposes murine hematopoietic stem cells to malignant transformation. Blood.

[157] Trowbridge JJ, Orkin SH (2012). Dnmt3a silences hematopoietic stem cell self-renewal. Nat Genet.

[158] Sen GL, Reuter JA, Webster DE, Zhu L, Khavari PA (2011). DNMT1 maintains progenitor function in self-renewing somatic tissue. Nature.

[159] Sun D (2014). Epigenomic profiling of young and aged HSCs reveals concerted changes during aging that reinforce self-renewal. Cell Stem Cell.

[160] Li Z (2011). Deletion of Tet2 in mice leads to dysregulated hematopoietic stem cells and subsequent development of myeloid malignancies. Blood.

[161] Ko M (2011). Ten-Eleven-Translocation 2 (TET2) negatively regulates homeostasis and differentiation of hematopoietic stem cells in mice. Proc Natl Acad Sci U S A.

[162] Moran-Crusio K (2011). Tet2 loss leads to increased hematopoietic stem cell self-renewal and myeloid transformation. Cancer Cell.

[163] Quivoron C (2011). TET2 inactivation results in pleiotropic hematopoietic abnormalities in mouse and is a recurrent event during human lymphomagenesis. Cancer Cell.

[164] Mikkelsen TS (2007). Genome-wide maps of chromatin state in pluripotent and lineage-committed cells. Nature.

[165] Li H (2017). Polycomb-like proteins link the PRC2 complex to CpG islands. Nature.

[166] Kamminga LM (2006). The polycomb group gene Ezh2 prevents hematopoietic stem cell exhaustion. Blood.

[167] Hidalgo I (2012). Ezh1 is required for hematopoietic stem cell maintenance and prevents senescence-like cell cycle arrest. Cell Stem Cell.

[168] Iwama A (2004). Enhanced self-renewal of hematopoietic stem cells mediated by the polycomb gene product Bmi-1. Immunity.

[169] Kinkel SA (2015). Jarid2 regulates hematopoietic stem cell function by acting with polycomb repressive complex 2. Blood.

[170] Xie H (2014). Polycomb repressive complex 2 regulates normal hematopoietic stem cell function in a developmental-stage-specific manner. Cell Stem Cell.

[171] Lee SC (2015). Polycomb repressive complex 2 component Suz12 is required for hematopoietic stem cell function and lymphopoiesis. Blood.

[172] Kerenyi, M. A. et al. Histone demethylase Lsd1 represses hematopoietic stem and progenitor cell signatures during blood cell maturation. eLife doi:10.7554/eLife.00633 (2013). 10.7554/eLife.00633 PMC3687337 23795291

[173] Cellot S (2013). RNAi screen identifies Jarid1b as a major regulator of mouse HSC activity. Blood.

[174] Thieme S (2013). The histone demethylase UTX regulates stem cell migration and hematopoiesis. Blood.

[175] Djeghloul D (2016). Age-associated decrease of the histone methyltransferase SUV39H1 in HSC perturbs heterochromatin and B lymphoid differentiation. Stem cell reports.

[176] Katsumoto T (2006). MOZ is essential for maintenance of hematopoietic stem cells. Genes Dev.

[177] Perez-Campo FM, Borrow J, Kouskoff V, Lacaud G (2009). The histone acetyl transferase activity of monocytic leukemia zinc finger is critical for the proliferation of hematopoietic precursors. Blood.

[178] Sheikh BN (2016). MOZ (KAT6A) is essential for the maintenance of classically defined adult hematopoietic stem cells. Blood.

[179] Valerio DG (2017). Histone acetyltransferase activity of MOF is required for adult but not early fetal hematopoiesis in mice. Blood.

[180] Yu VWC (2016). Epigenetic memory underlies cell-autonomous heterogeneous behavior of hematopoietic stem cells. Cell.

[181] Maegawa S (2014). Age-related epigenetic drift in the pathogenesis of MDS and AML. Genome Res.

[182] Yu VWC (2017). Epigenetic memory underlies cell-autonomous heterogeneous behavior of hematopoietic stem cells. Cell.

[183] Taiwo O (2013). DNA methylation analysis of murine hematopoietic side population cells during aging. Epigenetics.

[184] Beerman I (2013). Proliferation-dependent alterations of the DNA methylation landscape underlie hematopoietic stem cell aging. Cell Stem Cell.

[185] Adolfsson J (2005). Identification of Flt3+ lympho-myeloid stem cells lacking erythro-megakaryocytic potential a revised road map for adult blood lineage commitment. Cell.

[186] Cabezas-Wallscheid N (2014). Identification of regulatory networks in HSCs and their immediate progeny via integrated proteome, transcriptome, and DNA methylome analysis. Cell Stem Cell.

[187] Kramer A, Challen GA (2017). The epigenetic basis of hematopoietic stem cell aging. Semin Hematol.

[188] Grover A (2016). Single-cell RNA sequencing reveals molecular and functional platelet bias of aged haematopoietic stem cells. Nat Commun.

[189] Montecino-Rodriguez E (2019). Lymphoid-biased hematopoietic stem cells are maintained with age and efficiently generate lymphoid progeny. Stem Cell Rep.

[190] Ho YH (2019). Remodeling of bone marrow hematopoietic stem cell niches promotes myeloid cell expansion during premature or physiological aging. Cell Stem Cell.

[191] Yu VW, Scadden DT (2016). Heterogeneity of the bone marrow niche. Curr Opin Hematol.

[192] Calvi LM (2003). Osteoblastic cells regulate the haematopoietic stem cell niche. Nature.

[193] Zhang J (2003). Identification of the haematopoietic stem cell niche and control of the niche size. Nature.

[194] Guidi N (2017). Osteopontin attenuates aging-associated phenotypes of hematopoietic stem cells. EMBO J.

[195] Haylock DN (2007). Hemopoietic stem cells with higher hemopoietic potential reside at the bone marrow endosteum. Stem Cells.

[196] Kunisaki Y (2013). Arteriolar niches maintain haematopoietic stem cell quiescence. Nature.

[197] Ding L, Saunders TL, Enikolopov G, Morrison SJ (2012). Endothelial and perivascular cells maintain haematopoietic stem cells. Nature.

[198] Itkin T (2016). Distinct bone marrow blood vessels differentially regulate haematopoiesis. Nature.

[199] Bruns I (2014). Megakaryocytes regulate hematopoietic stem cell quiescence through CXCL4 secretion. Nat Med.

[200] Nakamura-Ishizu A, Takubo K, Fujioka M, Suda T (2014). Megakaryocytes are essential for HSC quiescence through the production of thrombopoietin. Biochem Biophys Res Commun.

[201] Zhao M (2014). Megakaryocytes maintain homeostatic quiescence and promote post-injury regeneration of hematopoietic stem cells. Nat Med.

[202] Heazlewood SY (2013). Megakaryocytes co-localise with hemopoietic stem cells and release cytokines that up-regulate stem cell proliferation. Stem Cell Res.

[203] Mendez-Ferrer S (2010). Mesenchymal and haematopoietic stem cells form a unique bone marrow niche. Nature.

[204] Zhao, M. et al. N-Cadherin-expressing bone and marrow stromal progenitor cells maintain reserve hematopoietic stem cells. Cell Rep 26, 652–669, doi:10.1016/j.celrep.2018.12.093 (2019). 10.1016/j.celrep.2018.12.093 PMC6890378 30650358

[205] Boyerinas B (2013). Adhesion to osteopontin in the bone marrow niche regulates lymphoblastic leukemia cell dormancy. Blood.

[206] Stier S (2005). Osteopontin is a hematopoietic stem cell niche component that negatively regulates stem cell pool size. J Exp Med.

[207] Nilsson SK (2005). Osteopontin, a key component of the hematopoietic stem cell niche and regulator of primitive hematopoietic progenitor cells. Blood.

[208] Haylock DN, Nilsson SK (2006). Osteopontin: a bridge between bone and blood. Br J Haematol.

[209] Zehentmeier S, Pereira JP (2019). Cell circuits and niches controlling B cell development. Immunol Rev.

[210] Fistonich C (2018). Cell circuits between B cell progenitors and IL-7(+) mesenchymal progenitor cells control B cell development. J Exp Med.

[211] Cordeiro Gomes, A. et al. Hematopoietic stem cell niches produce lineage-instructive signals to control multipotent progenitor differentiation. Immunity 45, 1219–1231, doi:10.1016/j.immuni.2016.11.004 (2016). 10.1016/j.immuni.2016.11.004 PMC5538583 27913094

[212] Balzano, M. et al. Nidogen-1 Contributes to the interaction network involved in Pro-B cell retention in the peri-sinusoidal hematopoietic stem cell niche. Cell reports 26, 3257–3271 e3258, doi:10.1016/j.celrep.2019.02.065 (2019). 10.1016/j.celrep.2019.02.065 30893599

[213] Greenbaum A (2013). CXCL12 in early mesenchymal progenitors is required for haematopoietic stem-cell maintenance. Nature.

[214] Hirata Y (2018). CD150(high) bone marrow tregs maintain hematopoietic stem cell quiescence and immune privilege via adenosine. Cell Stem Cell.

[215] Pierini A (2017). Foxp3(+) regulatory T cells maintain the bone marrow microenvironment for B cell lymphopoiesis. Nat Commun.

[216] Hirata Y, Kakiuchi M, Robson SC, Fujisaki J (2019). CD150(high) CD4 T cells and CD150(high) regulatory T cells regulate hematopoietic stem cell quiescence via CD73. Haematologica.

[217] Sugiyama T, Kohara H, Noda M, Nagasawa T (2006). Maintenance of the hematopoietic stem cell pool by CXCL12-CXCR4 chemokine signaling in bone marrow stromal cell niches. Immunity.

[218] Pitulescu ME (2017). Dll4 and Notch signalling couples sprouting angiogenesis and artery formation. Nat Cell Biol.

[219] Omatsu Y (2010). The essential functions of adipo-osteogenic progenitors as the hematopoietic stem and progenitor cell niche. Immunity.

[220] Hooper AT (2009). Engraftment and reconstitution of hematopoiesis is dependent on VEGFR2-mediated regeneration of sinusoidal endothelial cells. Cell Stem Cell.

[221] Kusumbe AP, Ramasamy SK, Adams RH (2014). Coupling of angiogenesis and osteogenesis by a specific vessel subtype in bone. Nature.

[222] Yue R, Zhou BO, Shimada IS, Zhao Z, Morrison SJ (2016). Leptin receptor promotes adipogenesis and reduces osteogenesis by regulating mesenchymal stromal cells in adult bone marrow. Cell Stem Cell.

[223] Spencer JA (2014). Direct measurement of local oxygen concentration in the bone marrow of live animals. Nature.

[224] Chow A (2011). Bone marrow CD169+ macrophages promote the retention of hematopoietic stem and progenitor cells in the mesenchymal stem cell niche. J Exp Med.

[225] Ludin A (2012). Monocytes-macrophages that express alpha-smooth muscle actin preserve primitive hematopoietic cells in the bone marrow. Nat Immunol.

[226] Casanova-Acebes M (2013). Rhythmic modulation of the hematopoietic niche through neutrophil clearance. Cell.

[227] McCabe A, MacNamara KC (2016). Macrophages: key regulators of steady-state and demand-adapted hematopoiesis. Exp Hematol.

[228] Winkler IG (2010). Bone marrow macrophages maintain hematopoietic stem cell (HSC) niches and their depletion mobilizes HSCs. Blood.

[229] Hur J (2016). CD82/KAI1 maintains the dormancy of long-term hematopoietic stem cells through interaction with DARC-expressing macrophages. Cell Stem Cell.

[230] Maryanovich M (2018). Adrenergic nerve degeneration in bone marrow drives aging of the hematopoietic stem cell niche. Nat Med.

[231] Katayama Y (2006). Signals from the sympathetic nervous system regulate hematopoietic stem cell egress from bone marrow. Cell.

[232] Mendez-Ferrer S, Battista M, Frenette PS (2010). Cooperation of beta(2)- and beta(3)-adrenergic receptors in hematopoietic progenitor cell mobilization. Ann N Y Acad Sci.

[233] Mendez-Ferrer S, Lucas D, Battista M, Frenette PS (2008). Haematopoietic stem cell release is regulated by circadian oscillations. Nature.

[234] Comazzetto S (2019). Restricted hematopoietic progenitors and erythropoiesis require scf from leptin receptor+ niche cells in the bone marrow. Cell Stem Cell.

[235] Zhou BO, Yue R, Murphy MM, Peyer JG, Morrison SJ (2014). Leptin-receptor-expressing mesenchymal stromal cells represent the main source of bone formed by adult bone marrow. Cell Stem Cell.

[236] Tikhonova AN (2019). The bone marrow microenvironment at single-cell resolution. Nature.

[237] Wolock SL (2019). Mapping Distinct Bone Marrow Niche Populations and Their Differentiation Paths. Cell Rep.

[238] Zhong, L. et al. Single cell transcriptomics identifies a unique adipose lineage cell population that regulates bone marrow environment. eLife, doi:10.7554/eLife.54695 (2020). 10.7554/eLife.54695 PMC7220380 32286228

[239] Baccin C (2020). Combined single-cell and spatial transcriptomics reveal the molecular, cellular and spatial bone marrow niche organization. Nat Cell Biol.

[240] Baryawno N (2019). A Cellular Taxonomy of the Bone Marrow Stroma in Homeostasis and Leukemia. Cell.

[241] Isern J (2013). Self-renewing human bone marrow mesenspheres promote hematopoietic stem cell expansion. Cell Rep.

[242] Ghazanfari R (2017). Human primary bone marrow mesenchymal stromal cells and their in vitro progenies display distinct transcriptional profile signatures. Sci. Rep.

[243] Pinho S (2013). PDGFRalpha and CD51 mark human nestin+ sphere-forming mesenchymal stem cells capable of hematopoietic progenitor cell expansion. J Exp Med.

[244] Sacchetti B (2007). Self-renewing osteoprogenitors in bone marrow sinusoids can organize a hematopoietic microenvironment. Cell.

[245] Chan CK (2009). Endochondral ossification is required for haematopoietic stem-cell niche formation. Nature.

[246] Mizoguchi T (2014). Osterix marks distinct waves of primitive and definitive stromal progenitors during bone marrow development. Dev Cell.

[247] Ambrosi TH (2017). Adipocyte accumulation in the bone marrow during obesity and aging impairs stem cell-based hematopoietic and bone regeneration. Cell Stem Cell.

[248] Breitbach M (2018). In vivo labeling by CD73 marks multipotent stromal cells and highlights endothelial heterogeneity in the bone marrow niche. Cell Stem Cell.

[249] Chen Q (2019). Apelin(+) endothelial niche cells control hematopoiesis and mediate vascular regeneration after myeloablative injury. Cell Stem Cell.

[250] Asada N (2017). Differential cytokine contributions of perivascular haematopoietic stem cell niches. Nat Cell Biol.

[251] Ding L, Morrison SJ (2013). Haematopoietic stem cells and early lymphoid progenitors occupy distinct bone marrow niches. Nature.

[252] Himburg HA (2018). Distinct bone marrow sources of pleiotrophin control hematopoietic stem cell maintenance and regeneration. Cell Stem Cell.

[253] Xu C (2018). Stem cell factor is selectively secreted by arterial endothelial cells in bone marrow. Nat Commun.

[254] Nakahara F (2019). Engineering a haematopoietic stem cell niche by revitalizing mesenchymal stromal cells. Nat Cell Biol.

[255] Butler JM (2010). Endothelial cells are essential for the self-renewal and repopulation of Notch-dependent hematopoietic stem cells. Cell Stem Cell.

[256] Li W (2003). Primary endothelial cells isolated from the yolk sac and para-aortic splanchnopleura support the expansion of adult marrow stem cells in vitro. Blood.

[257] Li W, Johnson SA, Shelley WC, Yoder MC (2004). Hematopoietic stem cell repopulating ability can be maintained in vitro by some primary endothelial cells. Exp Hematol.

[258] Seandel M (2008). Generation of a functional and durable vascular niche by the adenoviral E4ORF1 gene. Proc Natl Acad Sci U S A.

[259] Poulos MG (2015). Vascular platform to define hematopoietic stem cell factors and enhance regenerative hematopoiesis. Stem Cell Rep.

[260] Kobayashi H (2010). Angiocrine factors from Akt-activated endothelial cells balance self-renewal and differentiation of haematopoietic stem cells. Nat Cell Biol.

[261] Butler JM (2012). Development of a vascular niche platform for expansion of repopulating human cord blood stem and progenitor cells. Blood.

[262] Himburg HA (2012). Pleiotrophin regulates the retention and self-renewal of hematopoietic stem cells in the bone marrow vascular niche. Cell Rep.

[263] Doan PL (2013). Epidermal growth factor regulates hematopoietic regeneration after radiation injury. Nat Med.

[264] Heissig B (2002). Recruitment of stem and progenitor cells from the bone marrow niche requires MMP-9 mediated release of kit-ligand. Cell.

[265] Poulos MG (2013). Endothelial Jagged-1 is necessary for homeostatic and regenerative hematopoiesis. Cell Rep.

[266] Nolan DJ (2013). Molecular signatures of tissue-specific microvascular endothelial cell heterogeneity in organ maintenance and regeneration. Dev Cell.

[267] Langen UH (2017). Cell-matrix signals specify bone endothelial cells during developmental osteogenesis. Nat Cell Biol.

[268] Ramasamy SK, Kusumbe AP, Wang L, Adams RH (2014). Endothelial Notch activity promotes angiogenesis and osteogenesis in bone. Nature.

[269] Rafii S, Butler JM, Ding BS (2016). Angiocrine functions of organ-specific endothelial cells. Nature.

[270] Hellstrom M (2007). Dll4 signalling through Notch1 regulates formation of tip cells during angiogenesis. Nature.

[271] Leung A (2013). Uncoupling VEGFA functions in arteriogenesis and hematopoietic stem cell specification. Dev Cell.

[272] Sun L (2016). Angiopoietin-1 facilitates recovery of hematopoiesis in radiated mice. Am J Transl Res.

[273] Zhou, B. O., Ding, L. & Morrison, S. J. Hematopoietic stem and progenitor cells regulate the regeneration of their niche by secreting Angiopoietin-1. eLife **4**, e05521, doi:10.7554/eLife.05521 (2015). 10.7554/eLife.05521 PMC4411515 25821987

[274] Chen J (2020). Gli1(+) Cells couple with type H vessels and are required for Type H vessel formation. Stem Cell Rep.

[275] Shao L (2019). A Tie2-Notch1 signaling axis regulates regeneration of the endothelial bone marrow niche. Haematologica.

[276] Zhang J, Li L (2008). Stem cell niche: microenvironment and beyond. J Biol Chem.

[277] Moerman EJ, Teng K, Lipschitz DA, Lecka-Czernik B (2004). Aging activates adipogenic and suppresses osteogenic programs in mesenchymal marrow stroma/stem cells: the role of PPAR-gamma2 transcription factor and TGF-beta/BMP signaling pathways. Aging Cell.

[278] Kusumbe AP (2016). Age-dependent modulation of vascular niches for haematopoietic stem cells. Nature.

[279] Maryanovich, M., Takeishi, S. & Frenette, P. S. Neural regulation of bone and bone marrow. *Cold Spring Harbor Perspect Med* 8, doi:10.1101/cshperspect.a031344 (2018). 10.1101/cshperspect.a031344 PMC6119651 29500307

[280] Stolzing A, Jones E, McGonagle D, Scutt A (2008). Age-related changes in human bone marrow-derived mesenchymal stem cells: consequences for cell therapies. Mech Ageing Dev.

[281] Garcia-Prat L, Sousa-Victor P, Munoz-Canoves P (2013). Functional dysregulation of stem cells during aging: a focus on skeletal muscle stem cells. FEBS J.

[282] Siegel G (2013). Phenotype, donor age and gender affect function of human bone marrow-derived mesenchymal stromal cells. BMC Med.

[283] Wagner W (2009). Aging and replicative senescence have related effects on human stem and progenitor cells. PLoS ONE.

[284] Ganguly P (2019). The analysis of in vivo aging in human bone marrow mesenchymal stromal cells using colony-forming unit-fibroblast assay and the CD45(low)CD271(+) phenotype. Stem Cells Int.

[285] Kim M (2012). Age-related alterations in mesenchymal stem cells related to shift in differentiation from osteogenic to adipogenic potential: implication to age-associated bone diseases and defects. Mech Ageing Dev.

[286] Fazeli PK (2013). Marrow fat and bone–new perspectives. J Clin Endocrinol Metab.

[287] Paccou J, Hardouin P, Cotten A, Penel G, Cortet B (2015). The role of bone marrow fat in skeletal health: usefulness and perspectives for clinicians. J Clin Endocrinol Metab.

[288] Naveiras O (2009). Bone-marrow adipocytes as negative regulators of the haematopoietic microenvironment. Nature.

[289] Visnjic D (2004). Hematopoiesis is severely altered in mice with an induced osteoblast deficiency. Blood.

[290] Zhu J (2007). Osteoblasts support B-lymphocyte commitment and differentiation from hematopoietic stem cells. Blood.

[291] Martin SK (2018). mTORC1 plays an important role in osteoblastic regulation of B-lymphopoiesis. Sci Rep.

[292] Wu JY (2008). Osteoblastic regulation of B lymphopoiesis is mediated by Gs{alpha}-dependent signaling pathways. Proc Natl Acad Sci U S A.

[293] Poulos MG (2017). Endothelial transplantation rejuvenates aged hematopoietic stem cell function. J Clin Invest.

[294] Xing, Z. et al. Increased hematopoietic stem cell mobilization in aged mice. Blood (2006). 10.1182/blood-2005-12-010272 PMC1895568 16741255

[295] Frisch BJ (2019). Aged marrow macrophages expand platelet-biased hematopoietic stem cells via Interleukin1B. JCI Insight.

[296] Hoffmann C, Leitz MR, Oberdorf-Maass S, Lohse MJ, Klotz KN (2004). Comparative pharmacology of human beta-adrenergic receptor subtypes–characterization of stably transfected receptors in CHO cells. Naunyn-Schmiedeberg's Arch Pharmacol.

[297] Pioli PD, Casero D, Montecino-Rodriguez E, Morrison SL, Dorshkind K (2019). Plasma cells are obligate effectors of enhanced myelopoiesis in aging bone marrow. Immunity.

[298] Flores RR (2017). Expansion of myeloid-derived suppressor cells with aging in the bone marrow of mice through a NF-kappaB-dependent mechanism. Aging Cell.

[299] Baratono SR, Chu N, Richman LP, Behrens EM (2015). Toll-like receptor 9 and interferon-gamma receptor signaling suppress the B-cell fate of uncommitted progenitors in mice. Eur J Immunol.

[300] Chen X (2017). Bone marrow myeloid cells regulate myeloid-biased hematopoietic stem cells via a histamine-dependent feedback loop. Cell Stem Cell.

[301] Valletta S (2020). Micro-environmental sensing by bone marrow stroma identifies IL-6 and TGFbeta1 as regulators of hematopoietic ageing. Nat Commun.

[302] Dang VD, Hilgenberg E, Ries S, Shen P, Fillatreau S (2014). From the regulatory functions of B cells to the identification of cytokine-producing plasma cell subsets. Curr Opin Immunol.

[303] Nombela-Arrieta C, Manz MG (2017). Quantification and three-dimensional microanatomical organization of the bone marrow. Blood Adv.

